# Conformal Imidazolium 1D Perovskite Capping Layer Stabilized 3D Perovskite Films for Efficient Solar Modules

**DOI:** 10.1002/advs.202204017

**Published:** 2022-11-13

**Authors:** Ruihao Chen, Hui Shen, Qing Chang, Ziheng Tang, Siqing Nie, Bili Chen, Tan Ping, Binghui Wu, Jun Yin, Jing Li, Nanfeng Zheng

**Affiliations:** ^1^ Pen‐Tung Sah Institute of Micro‐Nano Science and Technology Jiujiang Research Institute National & Local Joint Engineering Research Center of Preparation Technology of Nanomaterials Innovation Laboratory for Sciences and Technologies of Energy Materials of Fujian Province College of Chemistry and Chemical Engineering Xiamen University Xiamen 361005 China; ^2^ State Key Laboratory of Solidification Processing Center for Nano Energy Materials School of Materials Science and Engineering Northwestern Polytechnical University and Shaanxi Joint Laboratory of Graphene Xi'an 710072 China

**Keywords:** 1D/3D structure, damp‐heat stability, *N*,*N*′‐dialkylbenzimidazolium, perovskite solar module

## Abstract

Although the perovskite solar cells have been developed rapidly, the industrialization of perovskite photovoltaics is still facing challenges, especially considering their stability issues. Here, the new type of benzimidazolium salt, *N,N*′‐dialkylbenzimidazolium iodide, is proposed and functionalized to convert the three‐dimensional (3D) FACs‐perovskite films into one‐dimensional (1D) capping layer topped 1D/3D structure either in individual device or module levels. This conformal interface modulation demonstrates that not only can effectively stabilize FACs‐based perovskite films by inhibiting the lateral and vertical iodide diffusions in devices or modules, ensuring an excellent operation and environmental stability, but also provides an excellent charge transporting channel through the well‐designed 1D crystal structure. Consequently, efficient device performance with power conversion efficiency up to 24.3% is readily achieved. And the large‐area perovskite solar modules with high efficiency (19.6% for the active areas of 18 cm^2^) and long‐term stability (about 500 h in AM 1.5G illumination or about 1000 h under double‐85 conditions) are also successfully verified.

## Graphical abstract

Dimensional management for high‐performance perovskite solar modules: An ultrastable imidazolium‐based one‐dimensional (1D) perovskite structure is developed to passivate the three‐dimensional perovskite film toward the stability improvement of module. The 1D layer reduces interface defects and promotes the efficient charge transport. High efficiencies of 24.3% in 0.12 cm^2^‐area device and 19.6% in 36 cm^2^‐total‐area module are achieved with excellent operation/damp‐heat stabilities.

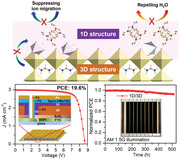

## 1 Introduction

Organic–inorganic hybrid perovskite materials have gained great attention for the next generation of photovoltaics attributed to their excellent optical and electronic properties, such as high absorption coefficient, long charge carrier diffusion length, and tunable bandgap.^[^ ^1^ ^]^ Although perovskite solar cells (PSCs) have made great progresses in power conversion efficiencies,^[^ ^2^ ^]^ it is still a challenge to overcome the bottleneck of stability issue. Besides the generally acknowledged thermodynamic instability for some perovskite composition, the surface metastable structures, such as the volatile organic ammonium components and kinds of defects, are indeed responsible for the low formation energy and low thermal stability of the metal‐halide perovskites.^[^ ^3^ ^]^ Consequently, the rapid degradation in materials and thus the decline in photovoltaic performance for PSC devices would occur when under kinds of environmental stresses or electric‐field driven during device operation. Additionally, the nonradiative recombination induced by the defects on the surface and grain boundaries of the perovskites would also limit the photovoltaic performance.^[^ ^4^ ^]^

Even in the encapsulated devices,^[^ ^5^ ^]^ the unfavorable ion diffusion that happened through the unstable surface and interface of perovskites would seriously impact the devices’ stability. Especially for perovskite modules, the ion diffusion not only occurs from the perovskite layer to the surface, known as the vertical diffusion but also happens in the inter‐connection area between the subcells, also called lateral diffusion. This lateral diffusion has been considered to be more critical for the module's stability in a large area. Although a few solutions have been reported to suppress the irreversible degradation caused by ion diffusion in PSC modules,^[^ ^6^ ^]^ such as Han et al. using nanomaterials as diffusion barrier to inhibit the ion migration and increase the module operational stability,^[^ ^7^ ^]^ there is still a lack of manipulation strategies based the device structure itself.

Constructing interface modification layer in devices, such as using porphyrin,^[^ ^8^ ^]^ metal‐clusters,^[^ ^9^ ^]^ or other organic molecules to passivate the surface defects and stabilize the perovskites,^[^ ^10^ ^]^ are well‐accepted measures. Among them, the application of two‐dimensional (2D) or one‐dimensional (1D) metal halide based materials shows the most promising prospect due to the simple doping or post‐treatment during the fabrication processes.^[^ ^11^ ^]^ For example, n‐butylammonium iodide, phenethylammonium iodide, and n‐octylammonium iodide^[^ ^12^ ^]^ have been successfully used to modify the three‐dimensional (3D) perovskite films to form stable 2D structures to passivate surface defects and improve the durability of PSCs. Other types of heterocyclic cations, including pyrrole, thiophene, imidazole, and guanidine^[^ ^13^ ^]^ are also used to treat the perovskites’ surface to form 1D structure to reduce the surface defects. For the current 1D or 2D structures, although they can avoid water and oxygen corrosion or metal/halide's ion chemical reactions,^[^ ^14^ ^]^ the ammonium salt species still need to be expanded and further developed to improve the PSC performance. Thereby, the development of multifunctional interface‐modulated materials, taking all account of the surface stabilization, defect passivation, and facilitating charge transport at the same time, are highly demanded.

With this motivation, an in situ constructed ultrastable 1D conformal capping layer‐based on imidazolium ions was developed in this work, and was used to stabilize the metal‐halide perovskite films by manipulating the interaction of its’ N group with [PbI_6_]^4−^ chain. A series of benzimidazolium salts, *N,N*′‐dialkylbenzimidazolium iodide, have been subtly designed and synthesized to treat the surface of FACsPbI_3_ perovskite films, yielding several kinds of 1D crystal structures with tunable electronic features. Benefitted from its ultrastable chemical properties and specific electronic structure, the optimized 1D structure helped to increase the moisture stability of the film, inhibit ion migration in devices, and enhance the operation stability under thermal and light stresses, while the efficient charges’ transportation still can be well maintained. By employing the 1D/3D hybrid films, a champion efficiency of 24.3% in the assembled PSCs was achieved. To the best of our knowledge, this excellent PCE is the highest performance for devices obtained using the 1D/3D hybrid structure. The unencapsulated PSCs presented super high light‐stability with maintaining 95% of its initial efficiency over 1400 h under continuous illumination in N_2_ atmosphere. Moreover, the large‐area 1D/3D‐based perovskite modules were also successfully fabricated by the blade‐coating method, achieving 19.6% and 18.0% efficiencies in the total areas of 36 cm^2^ (active area of 18 cm^2^) and 100 cm^2^ (active area of 56 cm^2^), respectively. The sealed PSC modules hold excellent light and damp‐heat stabilities (85 °C and 85% RH), demonstrating the great application potential of this new 1D structure in the perovskite photovoltaics.

## 2 Results and Discussion

**Figure 1:**
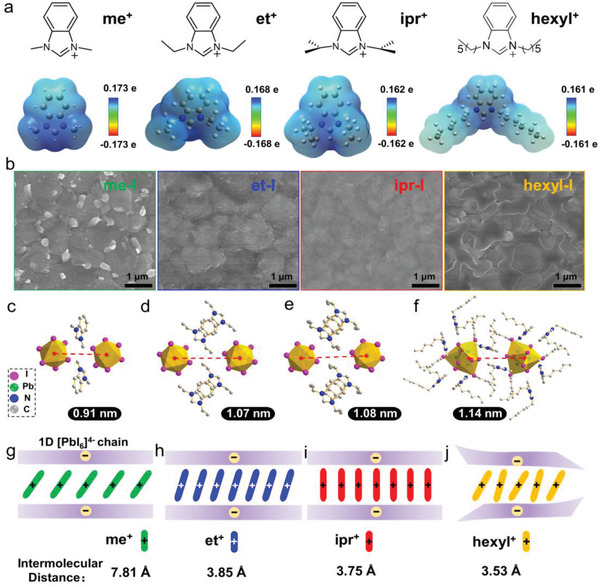
Investigation of the crystal and electronic structures for the 1D perovskite layer. a) Chemical structures and electrostatic surface potential (ESP) maps of me^+^, et^+^, ipr^+^, and hexyl^+^ cations (red indicates the electronegative part, blue indicates the electropositive part; blue atom is N, gray atom is C and white atom is H). b) Top‐view SEM images of perovskite films based on the treatments of me‐I, et‐I, ipr‐I, and hexyl‐I with the optimized concentrations. c–f) Front‐view crystal structures of 1D hybrids with c) mePbI_3_, d) etPbI_3_, e) iprPbI_3_, and f) hexylPbI_3_. The distance between two 1D chains is marked. g–j) Graphical illustration of 1D structures including g) mePbI_3_, h) etPbI_3_, i) iprPbI_3_, and j) hexylPbI_3_.

Construction and Analysis of the Conformal 1D Structure. Here, the conjugated *N,N*′‐dialkylbenzimidazolium salts were synthesized for the first time, and applied on the surface of perovskites to form the 1D passivation layer using a facile solution based post‐treatment (Figures S1–S5, Supporting Information). The adopted kinds of benzimidazolium salts, *N,N*‐dimethylbenzimidazolium iodide, *N,N*‐diethylbenzimidazolium iodide, *N,N*‐diisopropylbenzimidazolium iodide, and *N,N*‐dihexylbenzimidazolium iodide, were abbreviated as me‐I, et‐I, ipr‐I, and hexyl‐I for simplification, respectively. To optimize the structure by taking the charge transport performance into account, the Gaussian calculation was performed to obtain the electrostatic surface potential (ESP) of the four benzimidazole salts to study the local charge distribution around the molecules. As resolved from **Figure** **1**a, with the length of the carbon chain connected to N increases, the positive charge brought by N^+^ shows an improved uniformity on the surface. The obvious electrostatic potential value in the blue region means that this region is positive and electrophilic, and should be easier to obtain electrons.

Here, the smaller maximum value in ESP indicates a much more uniform surface potential distribution. Compared with the ESP values of me^+^ and et^+^ cations, the maximum ESP values of ipr^+^ and hexyl^+^ are smaller, at 0.162 and 0.161 e, respectively. Therefore, the surface potential distributions around ipr^+^ and hexyl^+^ ions are more uniform which would promote the charge transfer. As shown in Figure 1b; and Figure S5 (Supporting Information), due to the strong interaction between N group of *N,N*′‐dialkylbenzimidazolium and [PbI_6_]^4−^ chain, the original morphologies of the perovskite films have obviously changed and thus understandably new structures were formed on the surface by post‐treatment. Meantime, the additionally emerged X‐ray diffraction (XRD) peaks of the perovskite films in the low‐angle region clearly indicates the newly generated crystal layers (Figure S6, Supporting Information). Further single crystal X‐ray diffraction analysis revealed that all the surface capping layer held a 1D spatial structure, and the characteristic peaks in the simulated XRD patterns were consistent with these of the above experimental results (Figure S7, Supporting Information).

According to the crystal analysis shown in Figure 1c–f, APbI_3_ is composed of 1D chain [PbI_6_]^4−^ and surrounding A‐site cations. The size of the A‐site cations altered the spatial structure of Pb‐I chains (A is the *N,N*′‐dialkylbenzimidazolium salt). Correspondingly, the Pb‐I chains’ spacing has increased from 0.91 nm for mePbI_3_ to 1.07 nm for etPbI_3_, 1.08 nm for iprPbI_3_, and 1.14 nm for hexylPbI_3_. The results show that the larger size of the substituent, the larger of the 1D chain spacing can be obtained. Subsequently, in order to analyze the arrangement of A‐site cations between the 1D PbI_6_^4−^ chains, the simplified crystal structures (Figure S8 and Tables S1–S8, Supporting Information) were graphically illustrated in Figure 1g–j. In the structures of mePbI_3_, etPbI_3_, and iprPbI_3_, the A‐site cations are arranged in parallel between the 1D Pb‐I chains.

In the mePbI_3_ structure, the intermolecular distance of me^+^ ions is 7.81 Å, and the value obviously reduced to 3.85 Å for etPbI_3_, while much smaller (3.75 Å) for iprPbI_3_. However, as characterized by single crystal analysis, the formed hexylPbI_3_ structure is more complex and disordered. Due to the too long hexyl^+^ spatial structure in hexylPbI_3_, a serious deformation of the 1D Pb‐I structure would possibly happen, thus leading to the disorder and irregularity of the overall capping layer which might be not conducive to the effective surface passivation, although the intermolecular distance (3.53 Å) in hexylPbI_3_ is the smallest. Therefore, as the structure of iprPbI_3_ is more regular, with the parallelly arranged ipr^+^ molecules perpendicular to the 1D [PbI_6_]^4−^ chain which would facilitate the charge transfer, the iprPbI_3_ 1D structure was employed afterward for the passivation of perovskite films to improve both the photovoltaic and stability performances in corresponding devices.

**Figure 2:**
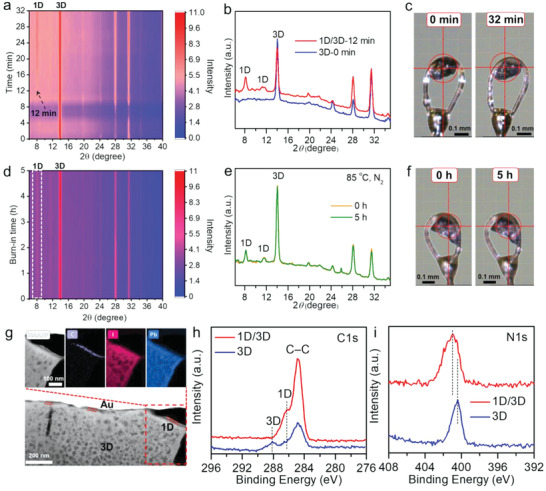
Crystallinity and structure evolution analysis of the 1D/3D perovskites. a) Contour maps of the in situ XRD patterns for the perovskite crystal converted from 3D to 1D/3D structure with the treatment of ipr‐I salt at different annealing durations (80 °C) in N_2_ atmosphere. b) XRD patterns of the perovskite crystals of 3D (0 min) and 1D/3D (12 min) structure. c) Digital photos of the perovskite crystals of 3D (0 min) and 1D/3D (32 min) structure. d) Contour maps of in situ XRD patterns for the 1D/3D perovskite crystal during the stability test under 85 °C in N_2_ atmosphere. e) XRD patterns and f) digital photos of the 1D/3D perovskite crystal annealed at 0 and 5 h. g) Cross‐sectional TEM images and EDS mapping of the 1D/3D perovskites. h,i) High‐resolution XPS spectra taken for h) C 1s and i) N 1s on the 3D and 1D/3D perovskite films.

In situ Formation of Conformal 1D/3D Structure. To further elucidate the in situ formation of the 1D structure on the surface of perovskite films, the single‐crystal XRD diffraction patterns of FACsPbI_3_ were used to monitor the evolution process (using a heating nitrogen flow as the heating source). Here, the iprPbI_3_ 1D structure was selected as a paradigm, and the presynthesized FA_0.9_Cs_0.1_PbI_3_ crystals are used as the 3D perovskites. The characterized XRD patterns and diffraction points are consistent with the previous work (Figures S9 and S10, Supporting Information).^[^ ^15^ ^]^ The contour maps of in situ XRD patterns were gathered at different annealing durations (**Figure** **2**a,b). The characteristic peaks located at 13.99°, 28.09°, and 31.48° can be assigned to the 3D perovskites. With increasing the annealing time to 12 min there was an appearance of 1D structure related peaks (8.13^o^ as a benchmark). And the 1D structure's diffraction peaks are consistent with the corresponding simulated XRD patterns (seen in Figure S10, Supporting Information). Interestingly, after the annealing for 32 min, the black color of perovskite crystal of 1D/3D structure was almost unchanged compared to that of the untreated 3D structure (Figure 2c).

To further investigate the stability of 1D/3D structure, contour maps of the in situ XRD patterns (Figure 2d) during the total 5 h of annealing for the 1D/3D perovskite crystal were plotted, together with the almost unchanged XRD patterns and digital photos Figure 2e,f, which clearly show the unchanged crystalline morphology of 1D/3D structure under 85 °C thermal condition. And in 85 °C and 60% RH condition, there are a large amount of PbI_2_ or *δ* phase in the 3D films, and the 1D/3D film maintained the pristine morphology after 50 h (Figure S11, Supporting Information). Meanwhile, additional characterization analysis indicated that the iprPbI_3_ 1D structure still can stabilize well even at 200 °C combined with excellent water stability (Figures S12 and S13, Supporting Information). The above results well suggested the in situ growth of the 1D structure and the excellent thermal and water resistance of the formed 1D/3D hybrid structure. High resolution transmission electron microscope (HRTEM) characterization further verified the as‐formed dense and uniform 1D film capped on the perovskite film (Figure 2g). As displayed in the enlarged region of energy dispersive spectrometer (EDS) mapping, the high ratio of carbon content in ipr^+^ (C_13_N_2_H_19_^+^) structure leads to a well‐defined carbon‐rich 1D layer on the film surface, and the distribution of I and Pb elements are uniformly distributed.

In order to study the surface chemical states in the as‐constructed 1D/3D structure, the X‐ray photoelectron spectroscopy (XPS) spectra of C 1s, N 1s, Pb 4f, and I 3d were carefully analyzed. The distribution of C 1s related C—C (contaminated carbon, 284.8 eV), 1D (N—C—N), and 3D (HC(NH_2_)_2_, FA^+^) peaks were well resolved from the low to high binding energies in the spectra (Figure 2h). The N 1s signal is mainly composed of a single peak with a binding energy of 400.4 eV, which can be attributed to the C=NH_2_^+^ bond of the FA cation. While after the surface 1D treatment, the binding energy of N 1s increased, which should be attributed to the N—C—N structure of the *N,N*′‐dialkylbenzimidazolium cation (Figure 2i). Compared with the 3D film, the Pb 4f and I 3d peaks of the 1D/3D film both shifted to the lower binding energies due to the longer Pb—I bond in 1D structure than that of the 3D structure (Figure S14, Supporting Information).^[^ ^16^ ^]^ Moreover, the etching‐depth dependent XPS spectra of the 1D/3D perovskite film also demonstrated the well‐defined 1D perovskite capping layer located on the surface of 3D perovskite film, coinciding with the result of EDS mappings (Figure S15, Supporting Information).

**Figure 3:**
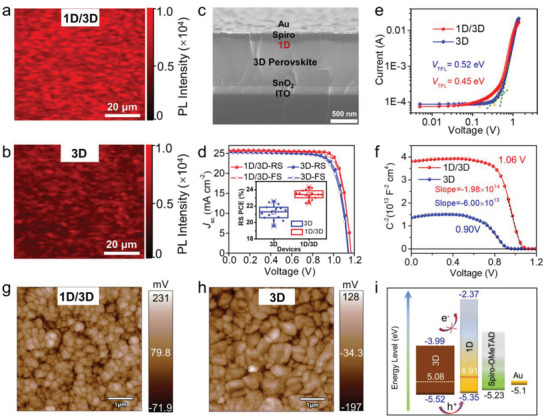
Photovoltaic performance and surface state of the 1D/3D structure. PL mapping of the 1D/3D film a) compared with 3D film b) in a large area. c) Cross‐sectional SEM image shows the device structure with 1D/3D film adopted for the champion cells. d) *J–V* curves of the 0.12 cm^2^ area PSCs with 3D and 1D/3D perovskite films based on FAPbI_3_ system. Inset is the RS PCE distribution of 15 individual PSCs based on 3D and 1D/3D films. e) Dark *J–V* curves of devices prepared using 3D and 1D/3D films for SCLC analysis. f) Mott*–*Schottky plots of the PSCs based on 3D and 1D/3D films at 1 kHz. Surface contact potential patterns of g) 1D/3D film compared with h) 3D film measured by KPFM. Inset are the corresponding topography images. i) Estimated energy level alignment of the PSCs based on 1D/3D films.

PV Performance Enhancement by with 1D Capping Layer. The in situ conformal passivating effect also can be intuitively seen in the steady‐state PL mapping (**Figure** **3**a,b), where uniformly improved fluorescence can be resolved on the 1D/3D film compared to these of the 3D film. It should be noticed that the morphology of the formed 1D film was apparently influenced by the concentration of the ipr‐I solution (Figure S16, Supporting Information). The ipr‐I concentrations have been optimized to be about 3 mg mL^−1^ during the post‐treatment according to the PL and UV–vis spectra and photovoltaic characterizations (Figures S17 and S18 and Table S9, Supporting Information). The FACs‐based PSCs in the n‐i‐p structure of ITO/SnO_2_/FA_0.9_Cs_0.1_PbI_3_/Spiro‐OMeTAD/Au by low‐temperature fabrication process were assembled to further assess the photovoltaic performance for the 1D/3D hybrid perovskite films (Figure 3c). The higher efficiencies (RS 21.50% and FS 21.32%) in the champion 1D/3D based devices were achieved than these of the control device (RS 19.91% and FS 19.03%) (Figure S19, Supporting Information).

To further improve the overall photovoltaic performance of PSCs, we used ipr‐I to post‐treat FAPbI_3_ perovskite film to construct the FAPbI_3_/1D based PSCs.^[^ ^2^, ^17^ ^]^ As shown in Figure 3d; and Table S10 (Supporting Information), the FAPbI_3_ device yields a champion PCE of 22.33% measured in the reverse scan, with *V*_oc_ of 1.14 V, *J*_sc_ of 25.39 mA cm^−2^, and FF of 76.75%. Notably, in the FAPbI_3_/1D PSC, the PCE is boosted to 24.43% with a large *V*_oc_ of 1.17 V, *J*_sc_ of 25.77 mA cm^−2^, and a remarkable FF of 80.70% with good reproducibility. The obtained short‐circuit current *J*_sc_ by *J–V* scan matched well with the extracted integrated value from the incident photon‐to‐electron conversion efficiency (IPCE) spectra (Figure S20, Supporting Information). To the best of our knowledge, this excellent PCE is among the highest performance for devices obtained using 1D/3D hybrid structure, as summarized in Table S11 (Supporting Information). In addition, the other optimized imidazolium derivatives have been used to fabricate PSCs which demonstrated the similar higher average PCEs than those of the untreated 3D devices, suggesting the universality of this imidazolium based 1D/3D devices (Figures S21–S24 and Table S12, Supporting Information). As resolved from the *J–V* curves, the increment of the PCEs is mainly credited to the *V*_oc_ and FF parameters which should be attributed to the reduced trap states and nonradiative recombination within the 1D/3D hybrid perovskite films based devices.^[^ ^18^ ^]^

Carrier Transport Improvement and Defect Passivation via 1D Structure. To better evaluate the passivation effect of 1D structure, the temperature dependent PL spectra (TDPL) were collected within the temperature range of 80–270 K (Figures S25 and S26, Supporting Information). The higher PL intensity and smaller blueshift in the emission peak from the 1D/3D film than those of the 3D films further confirmed the well‐eliminated defect states and suppressed nonradiative recombination.^[^ ^19^ ^]^ The detailed experiment including equations and fitting results were displayed in the Supporting Information figures. In the time‐resolved PL spectra (Figure S27, Supporting Information), the longer average decay time (*τ*) of 128.9 ns in 1D/3D film than that of 55.3 ns in the bare 3D system verified that the recombination loss was validly reduced after the 1D structure capping. Here, the defect density of the perovskite films was quantitatively evaluated by space‐charge‐limited current (SCLC) analysis based on the FTO/perovskite/Au configuration (Figure 3e). The trap‐filled limited voltage (*V*_TFL_) of the devices drew from the dark‐condition *J–V* curves was measured to be 0.45 V for the 1D/3D perovskite in contrast with 0.52 V for the 3D perovskite, producing the calculated trap densities of 4.5 × 10^15^ and 5.3 × 10^15^ cm^−3^, respectively. Understandably, the reduction of the defect density and suppressed nonradiative recombination have conducted to the improvement of *V*_oc_ and FF.^[^ ^20^ ^]^

As shown in Figure 3f, the built‐in potentials (*V*_bi_) in the both devices were assessed by Mott–Schottky analysis^[^ ^14^, ^21^ ^]^ indicating the extracted *V*_bi_ increased from 0.90 V in the 3D‐based device to 1.06 V in the 1D/3D‐based device. The higher slope of the 1D/3D device demonstrated the improved charge transfer occurred at the perovskite/HTL interface. Furthermore, the 1D layer also produced a more matched energy band alignment with the HTL in the device, as characterized by the Kelvin probe force microscopy (KPFM).^[^ ^22^ ^]^ As shown in Figure 3g,h; and Figure S28 (Supporting Information), the 1D structure capped on 3D film has reduced the surface roughness and improved the surface contact potential (SP). The increased SP value of 1D/3D film (from −34.3 to 79.8 mV) suggested that the Fermi level of perovskites’ surface became higher after the 1D passivation. Thus, this would boost the efficient holes transport in the cells and reduce the *V*_oc_ loss.^[^ ^22^, ^23^ ^]^ And, from ultraviolet photoelectron spectroscopy (UPS) measurements, the obtained valence band energy (*E*_VB_) of −5.35 for the 1D/3D film in comparison with −5.52 eV for the control 3D film (Figure S29, Supporting Information) indicates the potential better hole transfer in the 1D/3D device with the more matched energy level (Figure 3i).

**Figure 4:**
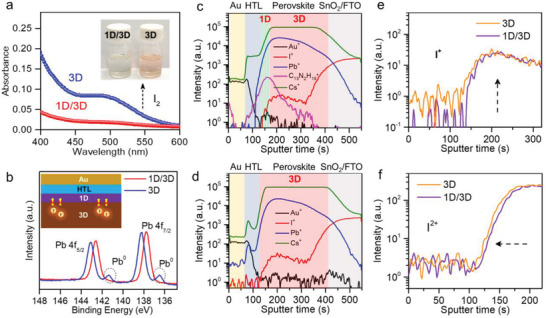
Stability investigation of the 1D/3D structure. a) UV–vis absorbance spectra recorded for the toluene solution taken from the vials containing 3D and 1D/3D films after 50 h. Inset are photographs of the toluene solution taken from the vials containing 3D and 1D/3D films after 50 h. b) High‐resolution XPS spectra of Pb 4f for the aged 1D/3D and 3D films being exposed to AM 1.5G illumination in N_2_ ambient for 50 h. (c) and (d) show the TOF‐SIMS spectra of the 1D/3D and control 3D devices after 100 h’ aging under 85 °C and AM 1.5G illumination, respectively. Enlarged‐regional TOF‐SIMS spectra for e) I^+^ and f) I^2+^ species of 1D/3D and 3D devices.

Suppressed Ion Diffusion by 1D Structure. In addition to improving the photovoltaic performance, the 1D structure can also effectively prevent the ion diffusion and stabilize the Pb‐I framework of the ionic perovskite, which is important for the long‐term stability.^[^ ^23^, ^24^ ^]^ To verify this inhibitory effect of the 1D passivating layer on the migration of I^−^ ions, the 1D/3D and 3D films were placed in the bottles filled with toluene and irradiated by the simulated AM 1.5G light at 60 °C, respectively. And the released pink‐colored I_2_ was monitored with comparison to the 3D perovskite film (Figure S30, Supporting Information). As shown in **Figure** **4**a, after 50 h, an obvious pink color was observed from the solution that contained the control 3D film. In comparison, the nearly unchanged colorless solution for the 1D/3D film was resolved. The corresponding absorption spectra also clearly indicates the more released amount of I_2_ from the 3D perovskite film than that of the 1D/3D film (Figure 4a). In addition, there was almost indistinguishable Pb^0^ appeared in the XPS spectra for the aged 1D/3D film, while evident Pb^0^ peak was observed in the aged 3D film (Figure 4b). The light stabilities of the 1D/3D and 3D films based PSCs were further explored in a N_2_ atmosphere as shown in Figure S31 (Supporting Information). The 1D/3D PSC showed an obvious stability improvement with maintaining 93.6% of the initial efficiency after over 1400‐h irradiation, while the 3D film‐based PSC dropped its efficiency to 67% of the pristine value after 500 h. Furthermore, the 1D/3D small area device also shows better stabilizing PCE output under MPP tracking and ≈60% RH without any encapsulation (Figure S32, Supporting Information).

Generally, the ions diffusion will be accelerated under light and high temperature aging environment. The time‐of‐flight secondary ion mass spectrometry (TOF‐SIMS) was used to probe the thermally (85 °C) aged devices after 100 h with continuous AM 1.5G illumination. In the 1D/3D device (Figure 4c), a bulky ipr^+^ cation (C_13_N_2_H_19_^+^) related signal peak from the 1D structure layer has been detected in the interface between the HTL (Spiro‐OMeTAD) and 3D perovskite layers. Compared with the aged 1D/3D device, an obvious distribution of Au element across the 3D perovskite‐based device confirmed the significant Au diffusion into the perovskite layer under the thermal and light aging (Figure 4d). With respect to the I ion signals, an increment by almost one order of magnitude was resolved in the bare 3D perovskite‐based device spatially shifting to the Spiro‐OMeTAD layer (Figure 4e,f). While for the device with 1D layer, the diffusion has been considerably suppressed. From the above results, it can be concluded that the benzimidazolium‐based 1D structure well help to stabilize the PSCs and effectively prevent the ions’ diffusion under different operation stresses.

**Figure 5:**
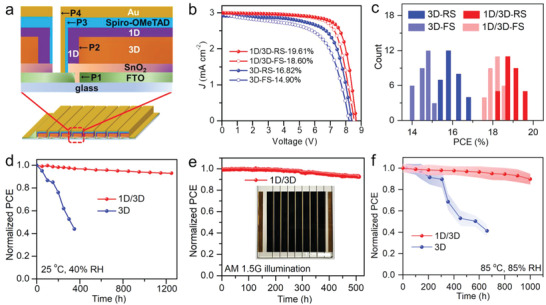
Photovoltaic performance of PSC modules. a) Schematic illustration of the module's configuration. b) *J–V* curves of the champion PSC modules (36 cm^2^) based on the 1D/3D and 3D perovskite films. c) Efficiency statistics of 30 individual modules based on 1D/3D and 3D perovskite films. d) PCE evolution of the unencapsulated PSC modules of 1D/3D and 3D under ambient condition (40% RH). e) Operation stability of the sealed 1D/3D structured module tracked at MPP under AM 1.5G illumination and 60% RH condition. Inset is the photo of the representative PSC module. f) Damp heat test of the sealed PSC modules based on 1D/3D perovskite films with PTAA HTL under 85 °C and 85% RH. The shaded area is the fluctuation range of the efficiency from five cells.

Efficient and Ultra‐stable PSC Modules with 1D/3D Structure. For the scalable fabrication of PSC modules, ion diffusion should be more seriously considered due to the additional lateral contact for the subcells, as illustrated in Figure S33 (Supporting Information). Therefore, the passivation process for perovskite modules has been designed to be done after the P2 scraping, producing a conformal coating of 1D layer both on the surface and side of the defined perovskite subcells (**Figure** **5**a; and Figure S34, Supporting Information). As this 1D layer was conformally grown by the solution based post‐treatment, the 1D structure can be uniformly and densely distributed on the top and side surface of the perovskite films crossing the whole large‐area module (36 cm^2^‐area), as characterized by the cross‐sectional scaning electronic microscopy (SEM) images (Figure S35, Supporting Information). And the ipr‐I post‐treatment help the side of the perovskites and residual PbI_2_ to transform into the 1D structure (Figure S36, Supporting Information). Apparently, this 1D structure would well inhibit the lateral and vertical iodide diffusion in the large‐area modules, as further evidenced by the elemental distribution analysis in the P2 channel after aging (Figure S37, Supporting Information).

The 36 cm^2^ n‐i‐p‐structured modules including eight subcells, which were separated and connected by straight and smooth P1–P4 scribing lines, have been fabricated using the 1D passivation method mentioned above (Figure S38, Supporting Information). As shown in Figure 5b, in the 1D/3D perovskite‐based module (18 cm^2^ of active area), a high PCE of RS 19.61% (FS 18.60%) was achieved, while the 3D module displayed lower efficiencies of RS 16.82% and FS 14.90% (Table S14, Supporting Information). And the average PCEs present a smaller hysteresis in the 1D/3D modules than these based on the 3D films (Figure 5c). Under storage condition with moderate relative humidity (40% RH), the unsealed 1D/3D structured module exhibited remarkably improved stability and kept 93% of their pristine efficiency over 1250 h, but the PCE of control module swiftly decayed to 44% of the original value after 350 h (Figure 5d). Furthermore, the operation stability of the sealed 1D/3D structured module could well maintain 92.4% of the origin efficiency over 510 h under the maximum power point (MPP) tracking with AM 1.5G irradiation at 60% RH (Figure 5e). While the PCE of the control module based on the 3D perovskite film rapidly declined to 66% of the origin PCE after 72 h, and a quick burn‐in degradation followed with a slight increase phenomenon in stability of sealed PSCs has been observed in previous reports (Figure S39, Supporting Information).^[^ ^10^, ^25^ ^]^ More stable PTAA^[^ ^26^ ^]^ was also adopted as the hole transport material (HTM) to test the damp‐heat stability of the sealed PSC modules (Figures S40 and S41 and Table S15, Supporting Information). Under the 85 °C and 85% RH condition, the sealed 1D/3D 36 cm^2^ area module showed an obvious augment and retained 90% of the initial value after 1000 h, while the 3D module rapidly decayed to 41% of the original efficiency after 660 h (Figure 5f). For a demonstration, much larger blade‐coated 100 cm^2^ area 1D/3D perovskite film with high uniformity (Figure S42, Supporting Information) has been produced and fabricated into modules which produced an attractive PCE of 18.0% efficiency (Figure S43 and Table S16, Supporting Information) with an active area of 56 cm^2^. The results well demonstrate the 1D/3D strategy have great potentials for the fabrication of stable and large‐area PSC modules.

## 3 Conclusion

In this work, an ultrastable benzimidazolium based 1D perovskite structure was introduced to passivate the perovskite film towards the stability improvement of perovskite solar devices/modules under different harsh stresses. The conformally grown 1D layer was facilely achieved by the solution based post‐treatment using the *N,N*′‐dialkylbenzimidazolium ions. Owing to the specific band structure and electronic properties, the conformal 1D coating layer not only demonstrates the effective passivation function on the interface defects, resulting in the low‐loss of open circuit voltage for the devices, but also promotes the efficient charge transport. Additionally, the produced excellent chemical stability within the interface of perovskites and HTL layer effectively prevents the ion migration in the vertical and horizontal directions under operation and thermal aging. Thus, efficient and stable perovskite modules were realized. The 6 × 6 cm^2^ (active area of 18 cm^2^) and 10 ×10 cm^2^ (active area of 56 cm^2^) solar modules achieved 19.6% and 18.0% efficiencies, respectively, with excellent operation/damp‐heat stabilities. Considering the facile in situ conformal growth, uniform conformal coverage, and excellent interface stability, this new type of passivation strategy based on the 1D structure is of great significance in the preparation of efficient and stable perovskite solar modules when toward the actual industrialization.

## 4 Experimental Section

### Materials

SnO_2_ colloids, chlorobenzene, CsI, PbI_2_, and CH_3_CN were purchased from Alfa Aesar. NMP, DMF, Benzimidazole, CH_2_Cl_2_, MgSO_4_, K_2_CO_3_, and IPA were purchased from SCRC. MACl, CsI, Li‐TFSI, and TBP were purchased from Xi'an P‐OLED. Formamidinium iodide (FAI), Spiro‐OMeTAD, ITO, and FTO glass (NSG, Tec‐7) were obtained from Advanced Election Technology Co.

### Synthesis of *N*,*N*′‐dialkylbenzimidazolium Iodide *N*,*N*‐dimethylbenzimidazolium Iodide (me‐I)

The compound was prepared according to method reported by Ong et al. with slight modification. Benzimidazole (20.0 mmol) and K_2_CO_3_ (22.0 mmol) were mixed in CH_3_CN (50 mL). After stirring the mixture for 2 h, methyl iodide (45.0 mmol) was added dropwise. The whole mixture was then heated at 85 °C for 3 d, and then quenched with water. The product was then extracted with CH_2_Cl_2_ (100 mL), and the obtained organic phase was dried over MgSO_4_ overnight. After drying up, the crude product was ultrasound over ethyl acetate and then dried under vacuum to gain the pale‐yellow powder (83% yield). *N,N*‐diethylbenzimidazolium iodide (et‐I), *N,N*‐diisopropylbenzimidazolium iodide (ipr‐I), and *N,N*‐dihexylbenzimidazolium iodide (hexyl‐I) were prepared in the similar procedure of me‐I.

### Solar Cell Fabrications

A compact SnO_2_ electron transport layer (ETL) on FTO or ITO was prepared by blade‐coating a SnO_2_ colloidal dispersion.^[^ ^27^ ^]^ For depositing of the FA_0.9_Cs_0.1_PbI_3_ perovskite layer, a mixture solution (0.1 mmol CsI, 0.90 mmol FAI, 1.00 mmol PbI_2_, additives (20 mg of MACl), 700 µL DMF and NMP (v: v = 9:1)) was spun on the as‐prepared substrate at 4000 rpm for 40 s, and then 100 µL chlorobenzene was dropped on the substrate prior the end of the process. Then, the substrate with perovskite precursor was heat‐treated at 150 °C for 30 min. The fabrication process of FAPbI_3_ film was referred to previous work.^[^ ^2^, ^17^ ^]^ For the ipr‐I post‐treated process, the ipr‐I IPA solution (1, 3, 5 mg mL^−1^) was blade‐coated on the perovskite film, followed by vacuum flash evaporation to remove the solution to ensure the uniformity of ipr‐I, and finally annealed at 80°C for 12 min. Hereafter, Spiro‐OMeTAD and Au electrode were deposited to finish the PSC production.^[^ ^23^, ^28^ ^]^ The formed Spiro‐OMeTAD HTL was about 100 nm and the thickness of Au electrode was controlled to be 60 nm during the thermal evaporation. The active area was defined by a 0.12 cm^2^ mask. The active area of the 36 cm^2^ (22.5 cm^2^ aperture area; 18.0 cm^2^ active area; GFF (geometrical fill factor) is 80%) and 100 cm^2^ (70.0 cm^2^ aperture area_;_ 56.0 cm^2^ active area; GFF is 80%) PSC modules were blade‐coating fabricated referring to the previous works.^[^ ^27^, ^29^ ^]^ PTAA polymer has been adopted for the damp‐heat stability testing of modules. The PTAA toluene solution (10 mg mL^−1^) containing 1.04 mg Li‐TFSI and 4 µL TBP was prepared, and then blade‐coated as the HTL. For the light stability and damp‐heat test, the encapsulation methods were referred to the previous reports.^[^ ^30^ ^]^

### Characterizations

*J–V* characteristics and incident photon‐to‐electron conversion efficiency (IPCE) spectra of PSCs were measured referring to previous work.^[^ ^31^ ^]^ The UV*–*vis spectra were recorded on a UV*–*vis spectrophotometer (PerkinElmer, LAMBDA 1050^+^). The XRD patterns, morphologies, steady and time‐resolved PL spectra and PL mapping measurements were carried out according to the previous report.^[^ ^32^ ^]^ The HRTEM and element‐mapping images of device were collected on a FEI Talos F200S transmission electron microscope.^[^ ^33^ ^]^ Trap state densities in perovskite films with the typical configuration devices (FTO/perovskite/Au) were measured by the SCLC model. The corresponding trap densities were calculated by employing the relationship between the trap density (*N*_trap_) and the trap‐filled limit voltage (*V*_TFL_): *V*_TFL_ = *qN*_trap_ *d* ^2^/2*εε*_0_ where *q* is the electronic charge, *d* is the thickness of the perovskite film, *ε*_0_ is the vacuum permittivity, and *ε* is the static dielectric constant of perovskite film. And the *V*_TFL_ were derived from the *J–V* curves.^[^ ^4^, ^34^ ^]^ To determine the ion migration in the aged devices, TOF‐SIMS (ION‐TOF GmbH, ToF SIMS V) was used. Depth profiling was accomplished with a O_2_ sputter ion gun and Bi primary‐ion gun. 280 × 280 µm^2^ of area were sputtered (1 keV O_2_^+^ sputter beam) and middle 133 × 133 µm^2^ of area were analyzed (30 keV Bi_3_^+^ primary ion beam). The Cs^+^ has high element sensitivity factor resulting in high peak intensity in TOF‐SIMS. For Pb signals, in the sputtering time 0–100s, Pb signal is interference. The real Pb signals of the perovskite layer existed in the sputtering time 100–400s.^[^ ^35^ ^]^ Ultraviolet photoelectron spectroscopy (UPS) measurement was performed on a PHI5000 Versa Probe III (Scanning ESCA Microprobe) instrument. Chemical surface states were performed on ESCALAB Xi+ X‐ray photoelectron spectrometer. Temperature‐dependent PL spectra is tested by Shimadzu IR350 spectrometer and ultraviolet laser excited by 193 nm laser excitation and laser power is 1 J cm^−2^. Chemical structures and electrostatic surface potential (ESP) of the *N,N*′‐dialkylbenzimidazolium salts were computed using the Gaussian 09 program by the B3LYP density functional theory (DFT) with 6‐311+G (2d, p) basis set. The ESP distributions were visualized using GaussView.

### X‐Ray Single‐Crystal Analysis

The diffraction data of the single crystals of compounds MePbI_3_, etPbI_3_, iprPbI_3_, and hexylPbI_3_ were collected on an X‐ray single‐crystal diffractometer (Rigaku Oxford Diffraction system) using Cu K*α* (*λ* = 1.54 184 Å) or Mo K*α* (*λ* = 0.71 073 Å) at 100 K. The data were processed using CrysAlisPro. The structures were solved and refined using Full‐matrix least‐squares based on F2 using ShelXT,^[^ ^36^ ^]^ ShelXL,^[^ ^37^ ^]^ in Olex2,^[^ ^38^ ^]^ and Shelxle.^[39]^ The thermal ellipsoids of the ORTEP diagram were done at 50% probability. The crystallographic data of mePbI_3_, etPbI_3_, iprPbI_3_, and hexylPbI_3_ are CCDC 2129781, CCDC 2129782, CCDC 2129783, and CCDC 2129784, respectively.

## Conflict of Interest

The authors declare no conflict of interest.

## Supporting information

Supporting Information

## Data Availability

The data that support the findings of this study are available from the corresponding author upon reasonable request.
