# Supplementary material for: Conformal Imidazolium 1D Perovskite Capping Layer Stabilized 3D Perovskite Films for Efficient Solar Modules
Source: Adv Sci (Weinh). 2022 Nov 13;9(36):2204017. doi: 10.1002/advs.202204017 (PMC9798973; doi:10.1002/advs.202204017)
Supplement: Supplementary file 1 — Supporting Information [file ADVS-9-2204017-s001.pdf]

ADVANCED SCIENCE
Open Access

Supporting Information

for *Adv. Sci.*, DOI 10.1002/advs.202204017

Conformal Imidazolium 1D Perovskite Capping Layer Stabilized 3D Perovskite Films for Efficient Solar Modules

Ruihao Chen, Hui Shen, Qing Chang, Ziheng Tang, Siqing Nie, Bili Chen, Tan Ping, Binghui Wu, Jun Yin, Jing Li* and Nanfeng Zheng*

Supporting Information

Conformal imidazolium 1D perovskite capping layer stabilized 3D perovskite films for efficient solar modules

Ruihao Chen,[#] Hui Shen,[#] Qing Chang,[#] Ziheng Tang, Siqing Nie, Bili Chen, Tan Ping, Binghui Wu, Jun Yin,[*] Jing Li,[*] Nanfeng Zheng

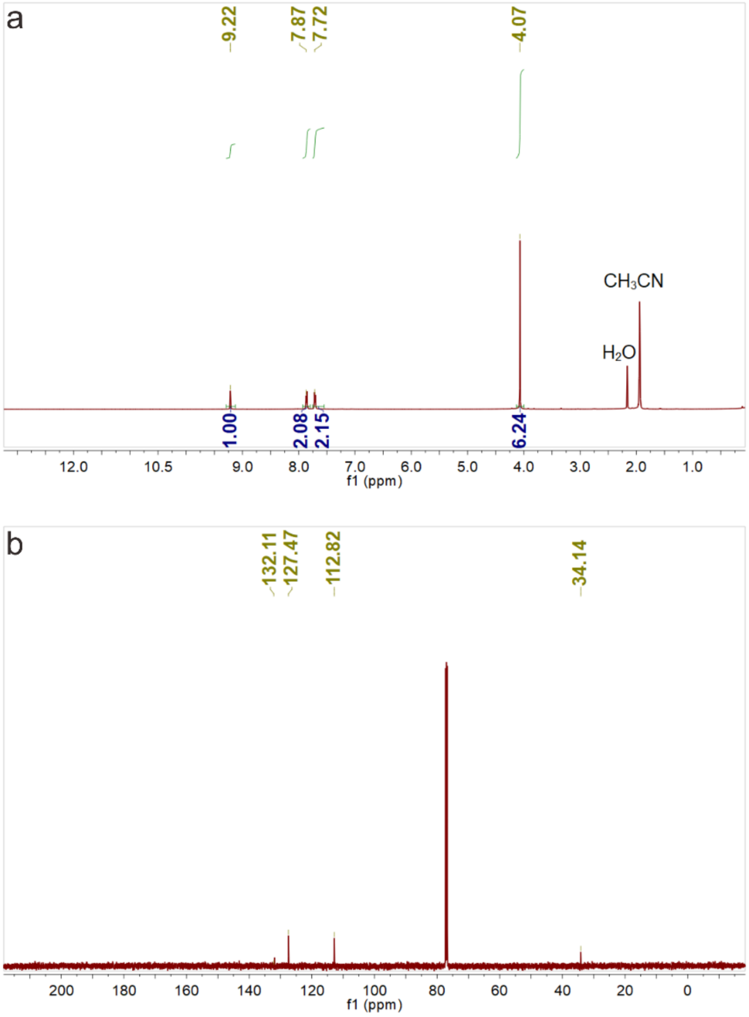

Figure S1. (a) ^{1}H and (b) ^{13}C NMR spectra of me-I powder.

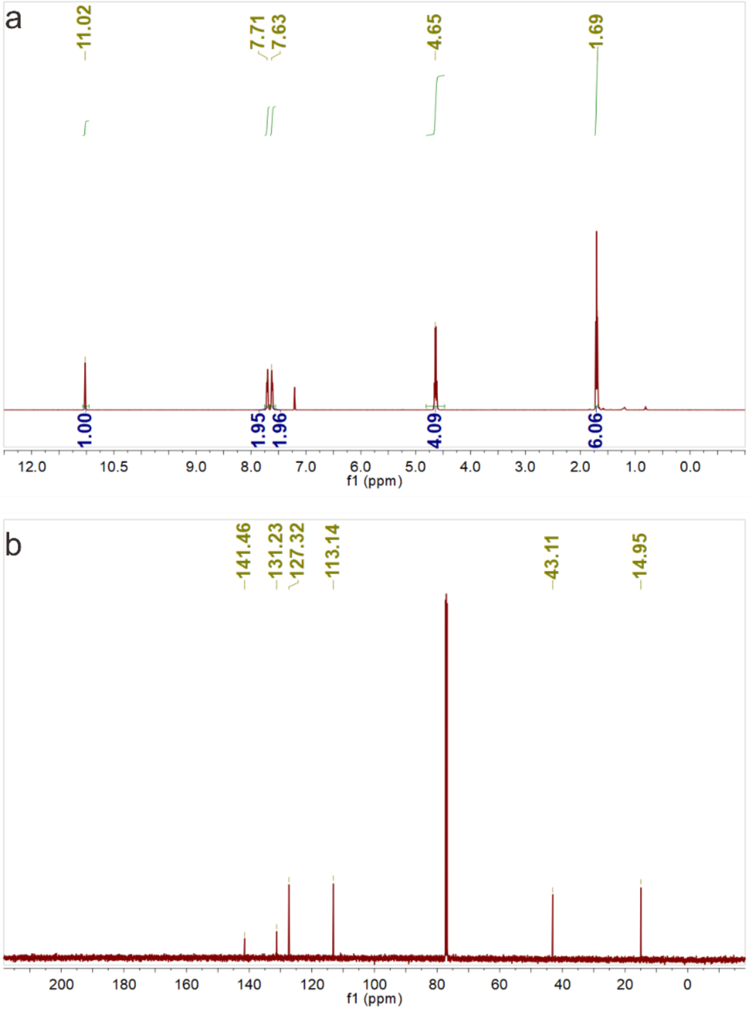

Figure S2. (a) ^{1}H and (b) ^{13}C NMR spectra of et-I powder.

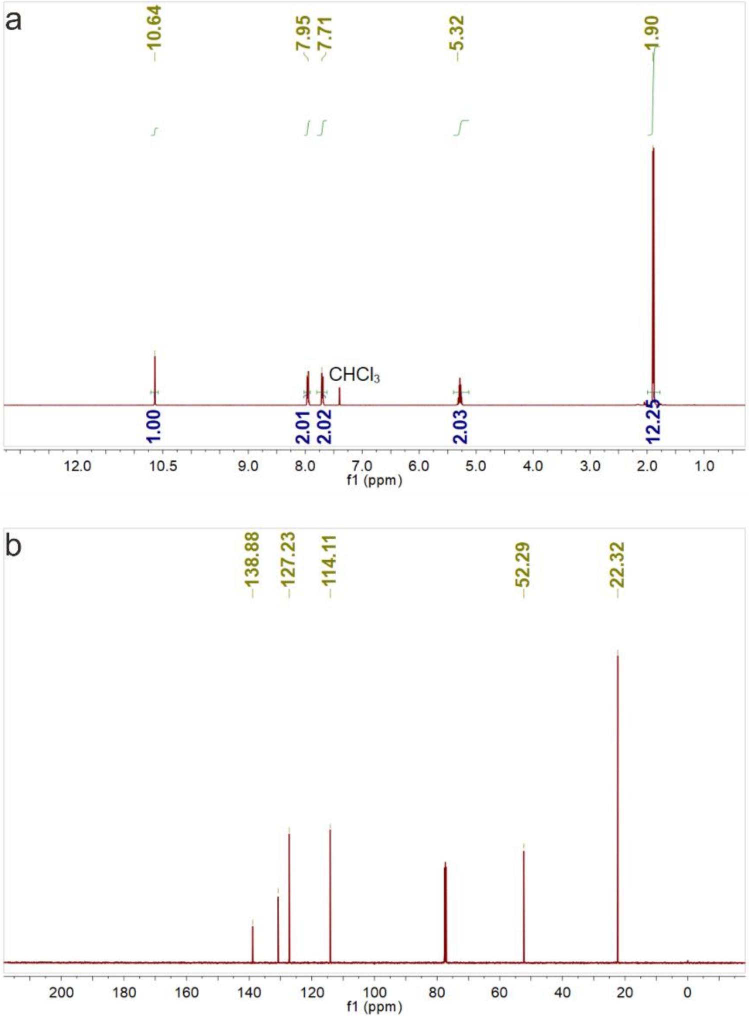

Figure S3. (a) ^{1}H and (b) ^{13}C NMR spectra of ipr-I powder.

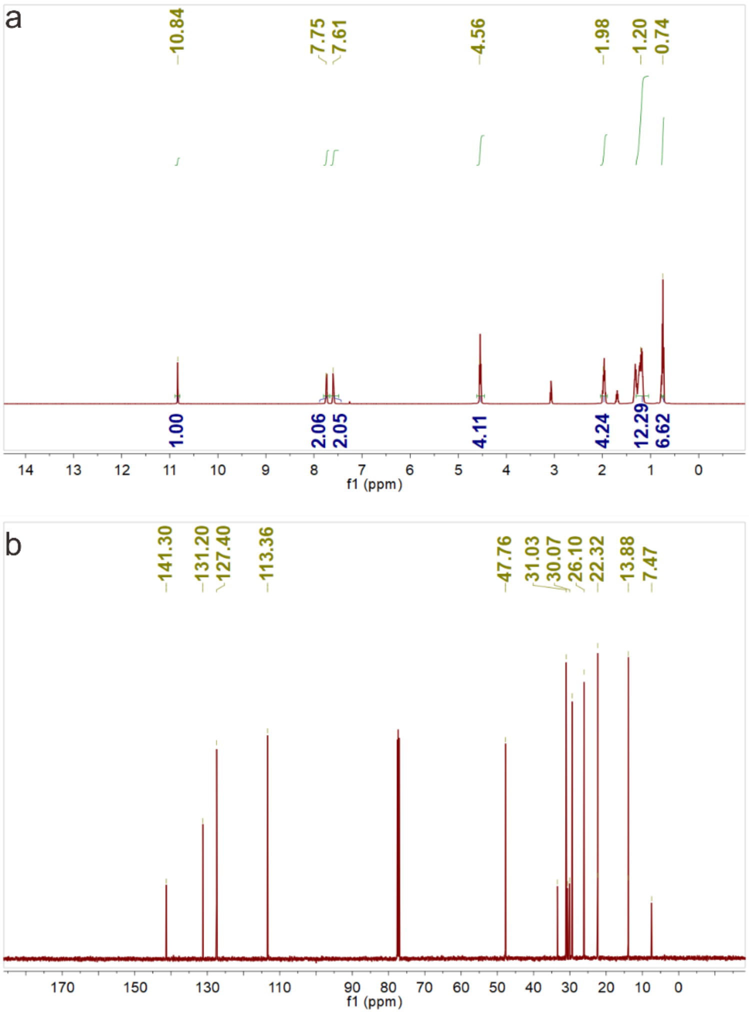

Figure S4. (a) ^{1}H and (b) ^{13}C NMR spectra of hexyl-I powder.

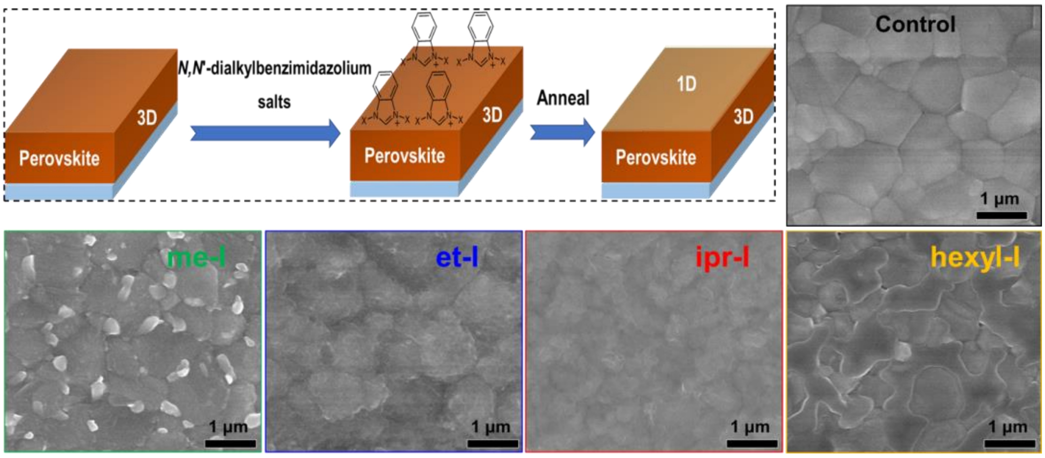

Figure S5. Schematic of *N,N'*-dialkylbenzimidazolium salts post-treatment on perovskite films and the formed 1D/3D film structure. SEM images of the control 3D perovskite film and series of perovskite films based on the treatments of me-I, et-I, ipr-I and hexyl-I with the optimized concentrations.

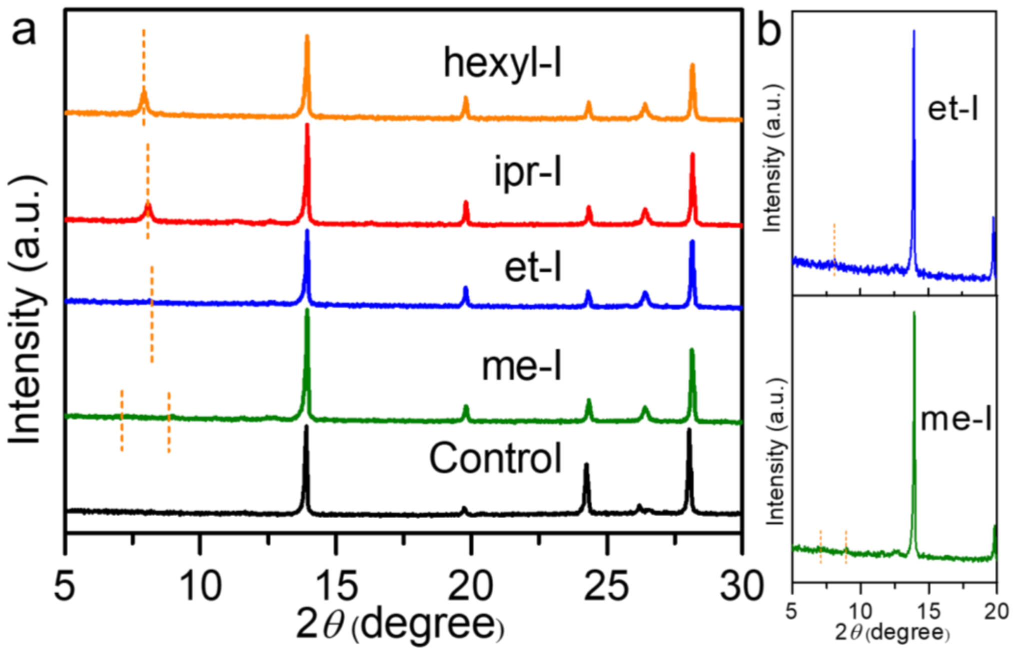

Figure S6. (a) XRD patterns of the control 3D perovskite film and series of perovskite films by the post treatments of me-I, et-I, ipr-I and hexyl-I, respectively. (b) Enlarged-regional XRD patterns of the perovskite films by the me-I and et-I post treatments.

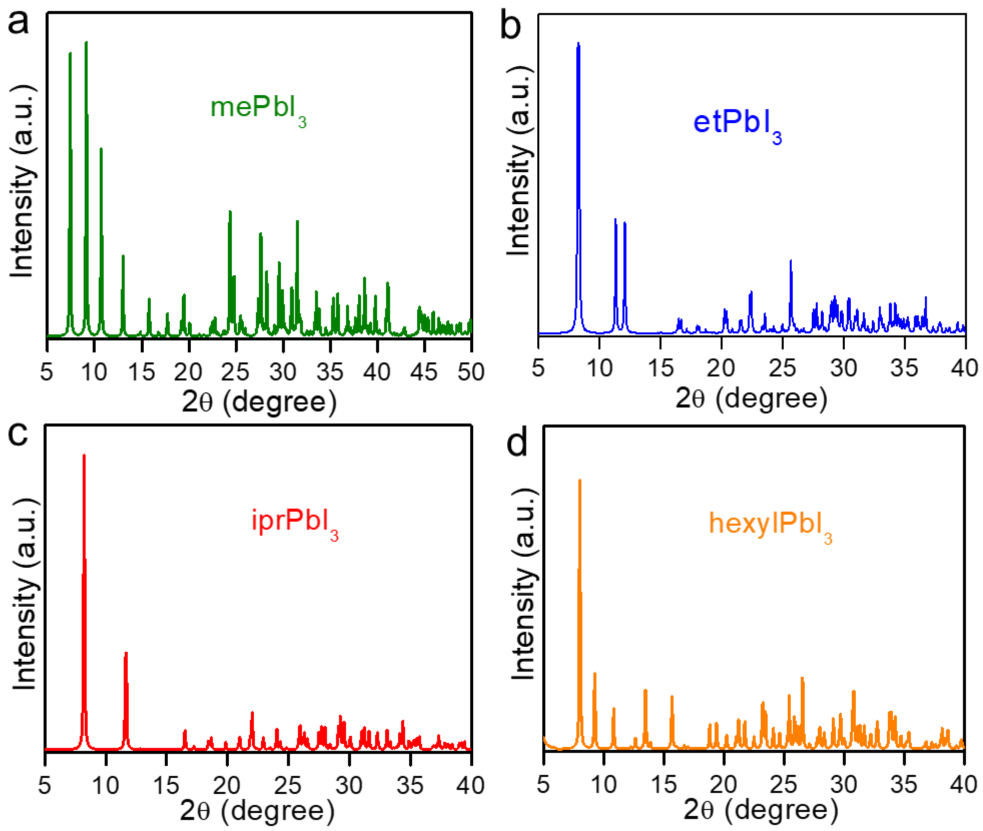

Figure S7. Simulated XRD patterns of the 1D perovskite structures. (a) $mePbI_3$, (b) $etPbI_3$, (c) $iprPbI_3$ and (d) $hexylPbI_3$.

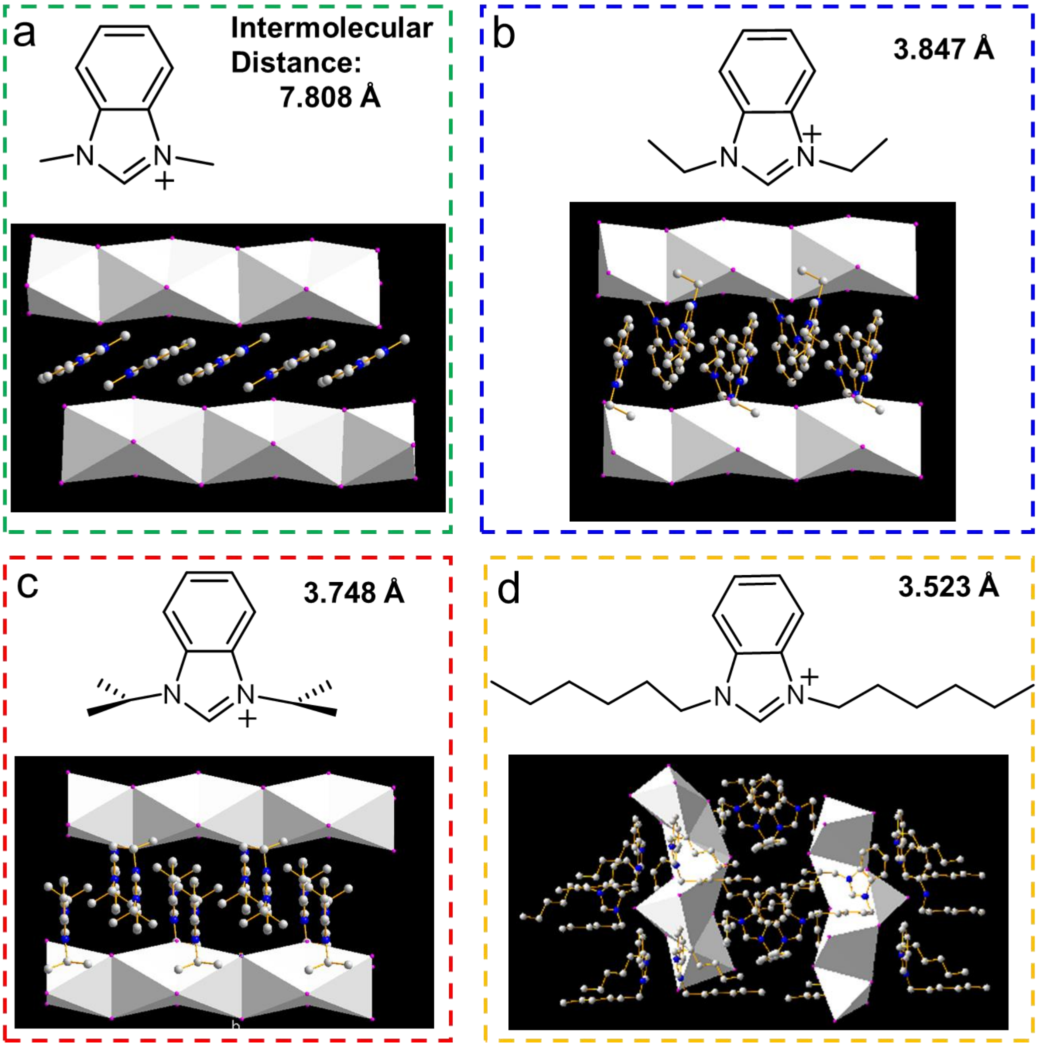

Figure S8. Side-view crystal structures of 1D hybrid structure. (a) $mePbI_3$, (b) $etPbI_3$, (c) $iprPbI_3$ and (d) $hexylPbI_3$. Inset are the π-π packing values. (Grey octahedron indicates the $[PbI_6]^{4-}$ unit, blue indicates the electropositive part; purple atom is I, blue atom is N and grey atom is C. Hydrogen atoms are omitted for clarity.)

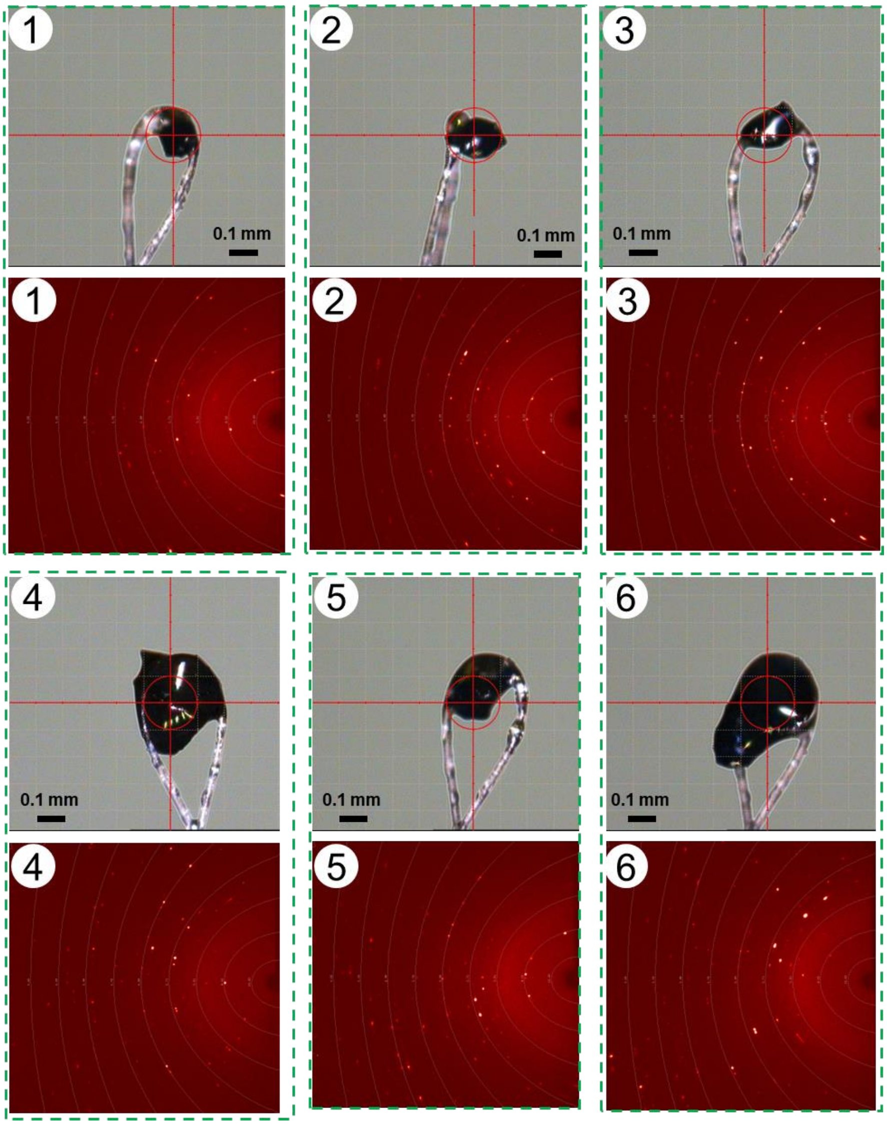

Figure S9. Digital pictures and diffraction points of the six individual $FACsPbI_3$ perovskite crystals. The Scale bar of perovskite crystals is 0.1 mm.

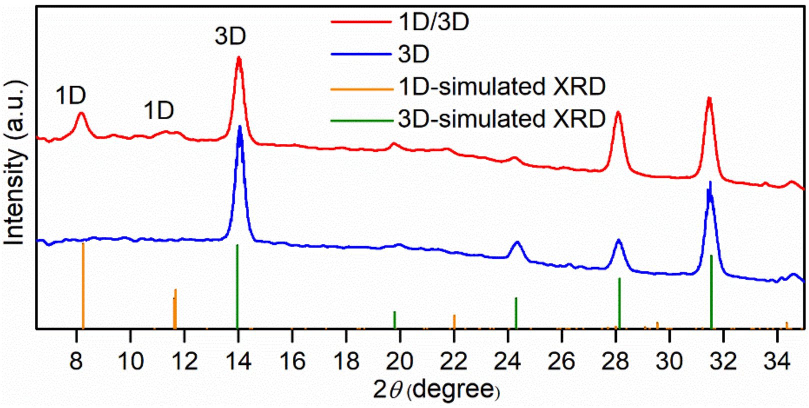

Figure S10. XRD patterns of 1D/3D and 3D perovskite crystals measured by single crystal diffractometer.

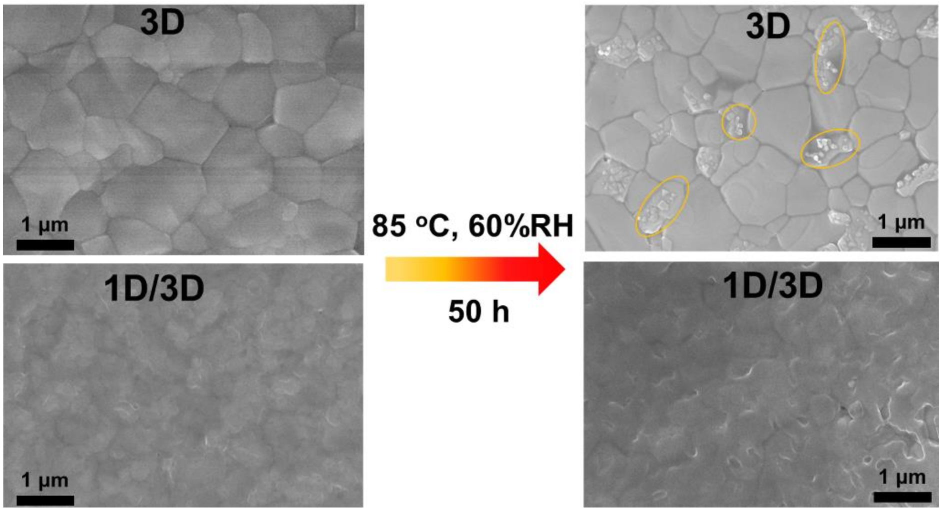

Figure S11. Damp-heat stability of 3D and 1D/3D films under 85 °C and 60% RH conditions. The yellow circles represent non-perovskite or PbI_2 phases.

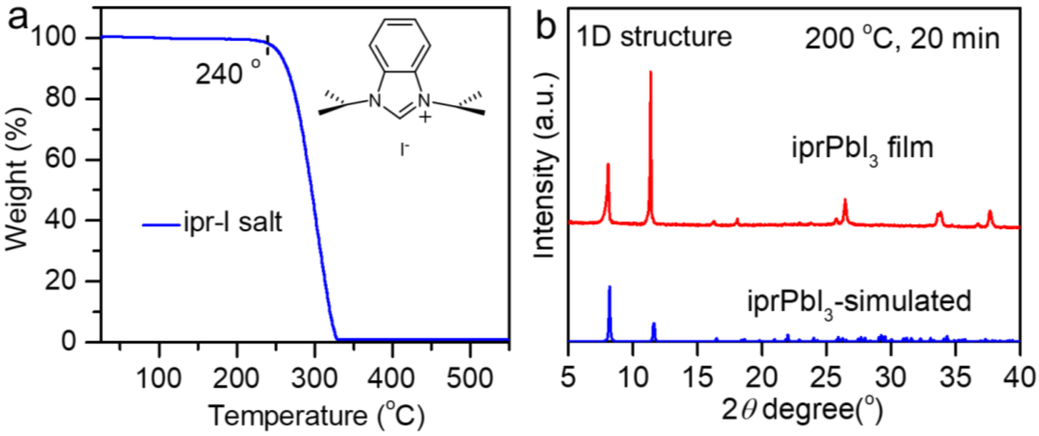

Figure S12. (a) TG analysis of ipr-I powder. (b) XRD patterns of $iprPbI_3$ film and the simulated XRD patterns of $iprPbI_3$ single crystal.

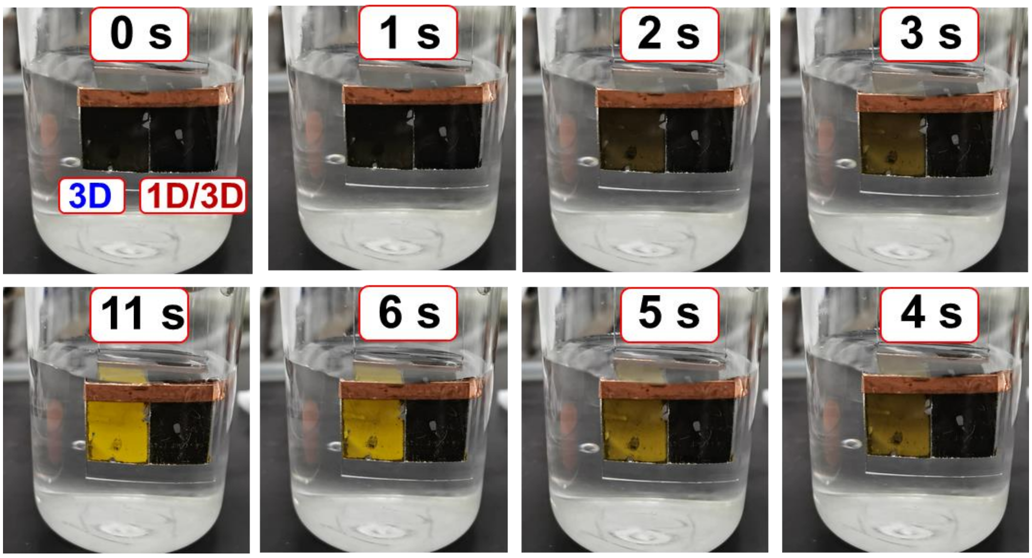

Figure S13. Water stability of the 1D/3D and 3D perovskite films. The 1D/3D and 3D films were immersed in water, and it can be observed that the 3D films immediately degraded or phase transformed within 2 seconds, but the 1D/3D films maintained black-colored perovskite phase even until 11th second showing the excellent moisture resistance.

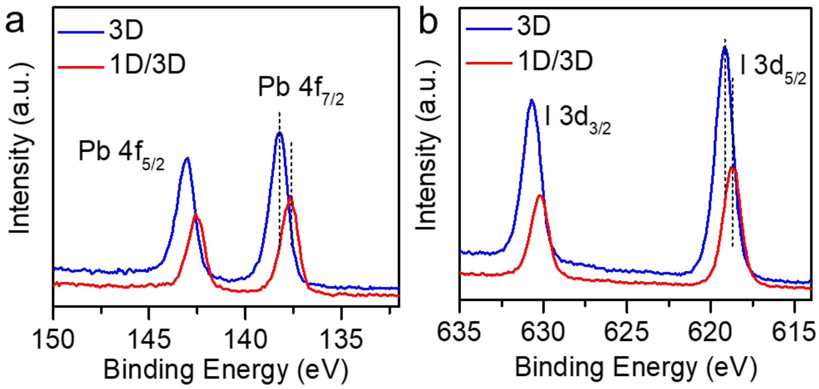

Figure S14. XPS spectra of Pb 4f and I 3d in the 1D/3D and 3D perovskite films.

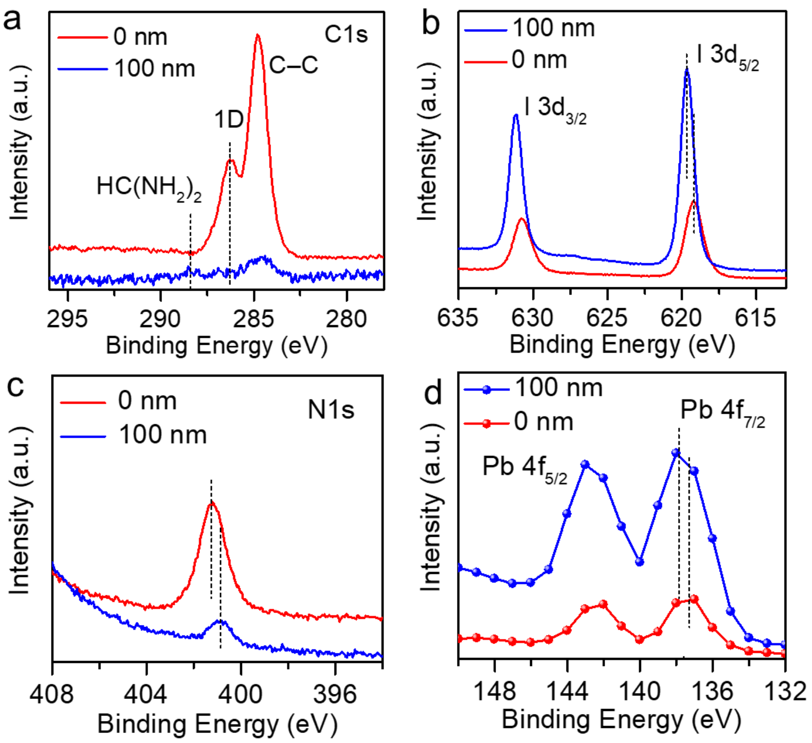

Figure S15. XPS depth related spectra of (a) C 1s, (b) I 3d, (c) N 1s and (d) Pb 4f in the 1D/3D perovskite films, with etching depth of 0 and 100 nm by etching 0 and 115 s, respectively. The depth-dependent XPS spectra of the 1D/3D perovskite film from the surface to the 3D inner were realized by etching for the different durations from 0 to 115 s. The peaks of C 1s, N 1s, I 3d and Pb 4f shifted and were consistent with the XPS results (Figure 2h-i and S13), and demonstrated again that the well-defined 1D layer capped on the surface of 3D perovskite film coinciding with the result of HRTEM-EDS mappings.

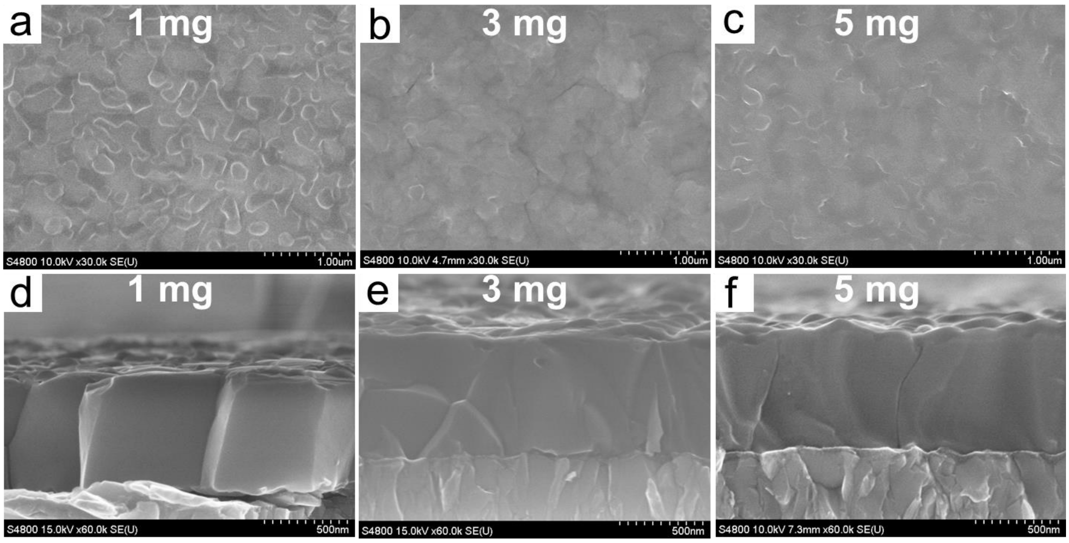

Figure S16. (a-c) Top-view and (d-f) cross-sectional SEM images of perovskite films post treated with 1, 3, and 5 mg/mL solutions. The results indicated that it is difficult to form an effective coverage if the ipr-I concentration is too low (1 mg/mL), while the higher concentration will easily lead to the rough surface of the film. The ipr-I concentration-dependent film growth rate for the 1D structure should be the main reason.

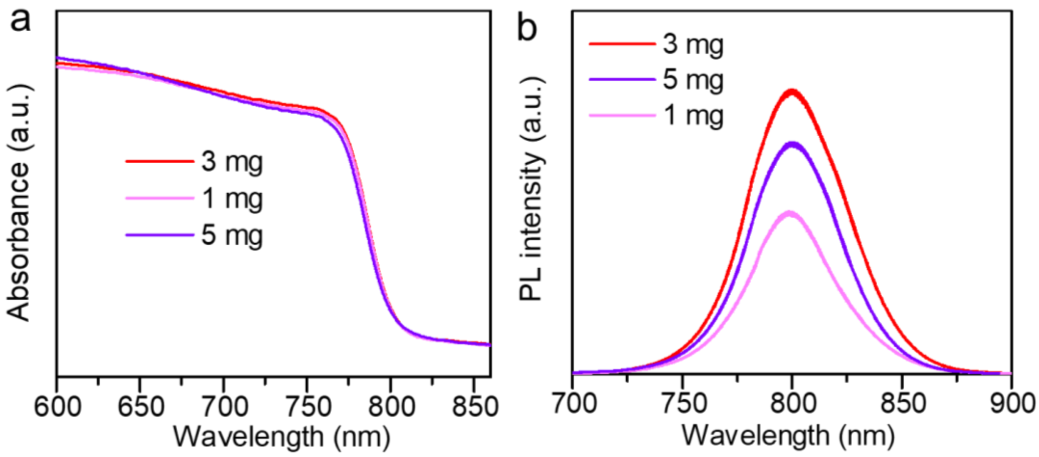

Figure S17 (a) UV-vis and (b) PL spectra of perovskite films post treated without or with 1, 3, and 5 mg/mL solutions.

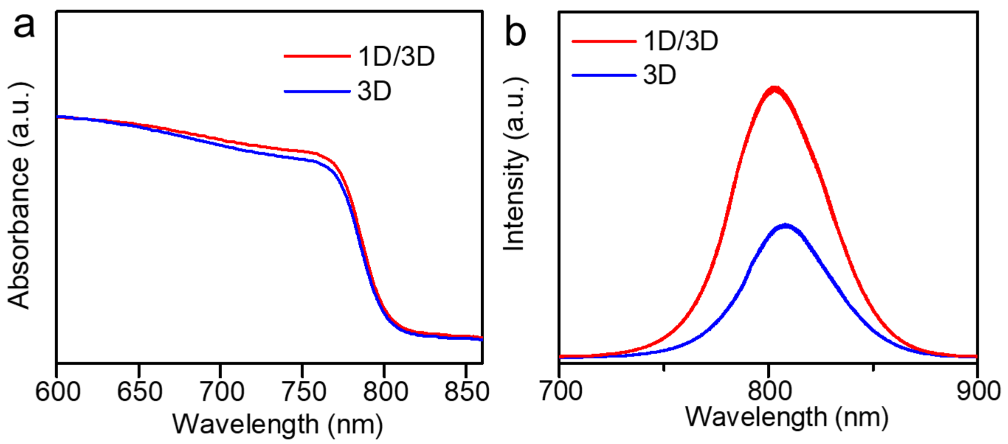

Figure S18. (a) UV-vis and (b) PL spectra of the 3D and 1D/3D perovskite films.

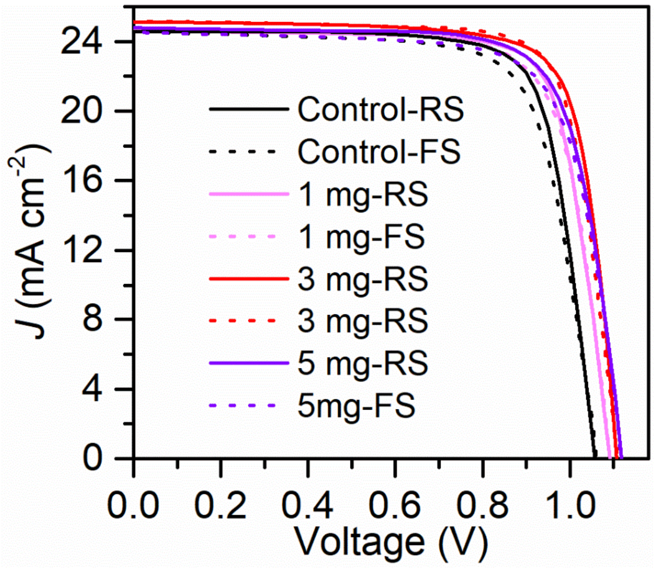

Figure S19. $J-V$ curves of PSCs based on perovskite films post treated without or with 1, 3, and 5 mg/mL solutions.

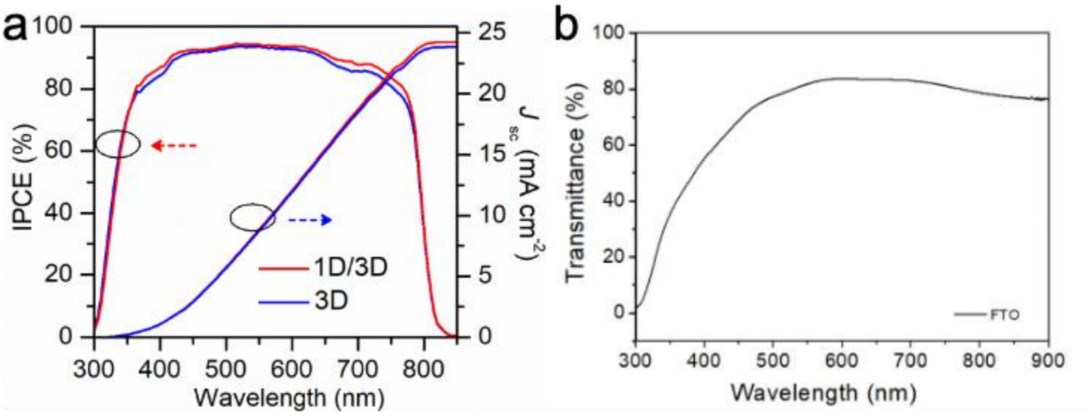

Figure S20. (a) Incident photon-to-electron conversion efficiency (IPCE) spectra and integrated J_{sc} of the champion devices with 1D/3D and 3D perovskite films based on $FAPbI_3$ system. The integrated J_{sc} from the IPCE spectrum for the PSCs with 1D/3D and 3D perovskite films was 24.25 and 23.82 mA cm^{-2}, respectively, which matched well with the values extracted from $J-V$ curves (~5.9% of deviation). (b) Transmittance of FTO substrate.

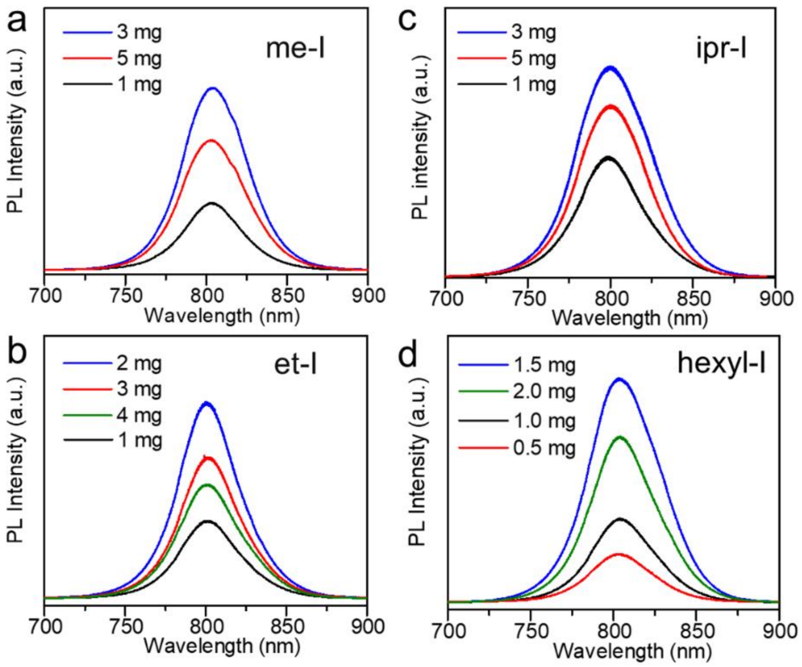

Figure S21. PL spectra of perovskite films based on the treatments of (a) me-I, (b) et-I, (c) ipr-I and (d) hexyl-I with different concentrations.

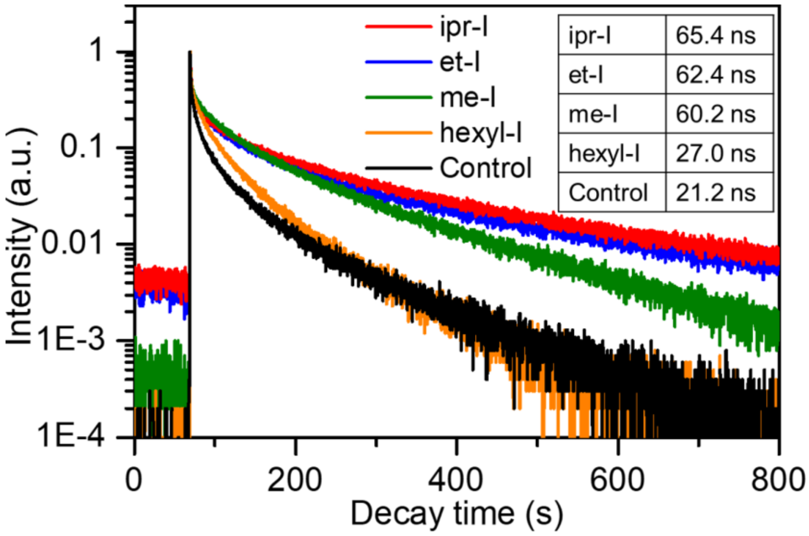

Figure S22. TRPL spectra of the perovskite films based on control and the optimized me-I, et-I, ipr-I and hexyl-I post-treatment process.

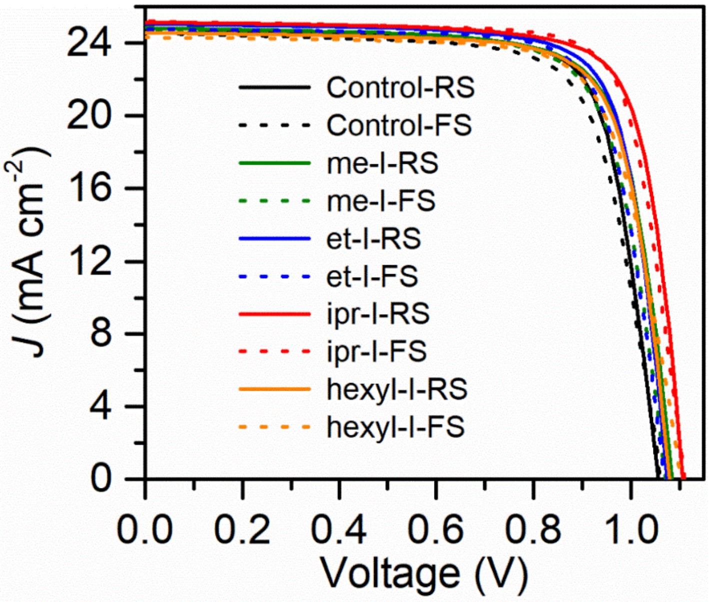

Figure S23. $J-V$ curves of the champion efficiencies of FACs-based PSCs based on control and the optimized me-I, et-I, ipr-I and hexyl-I post-treatment process.

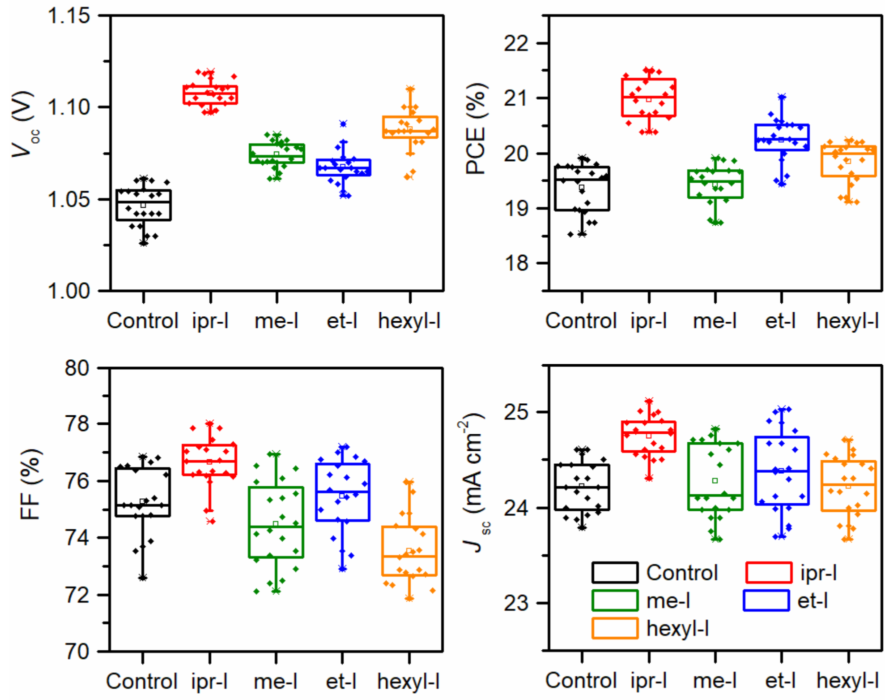

Figure S24. Distribution of 30 individual FACs-based PSCs based on the control, me-I, et-I, ipr-I and hexyl-I post-treated perovskite films.

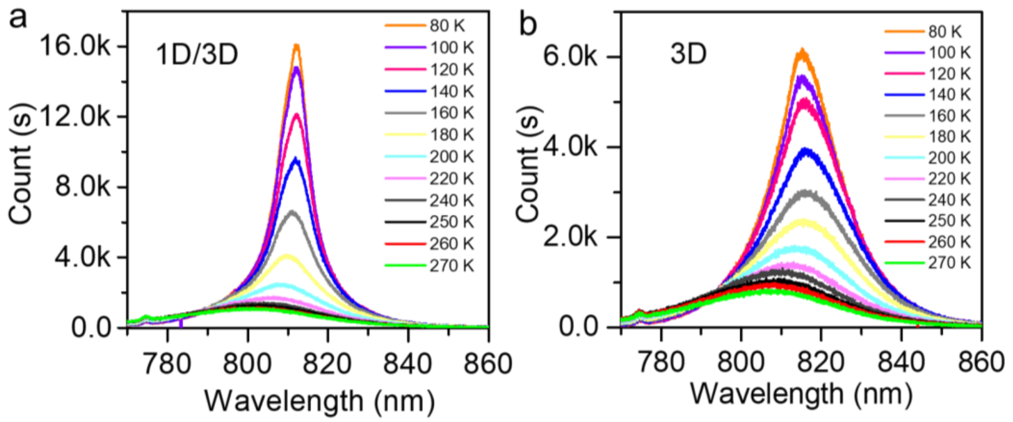

Figure S25. Temperature-dependent PL spectra of (a) 1D/3D and (b) 3D perovskite films from 80 to 270 K.

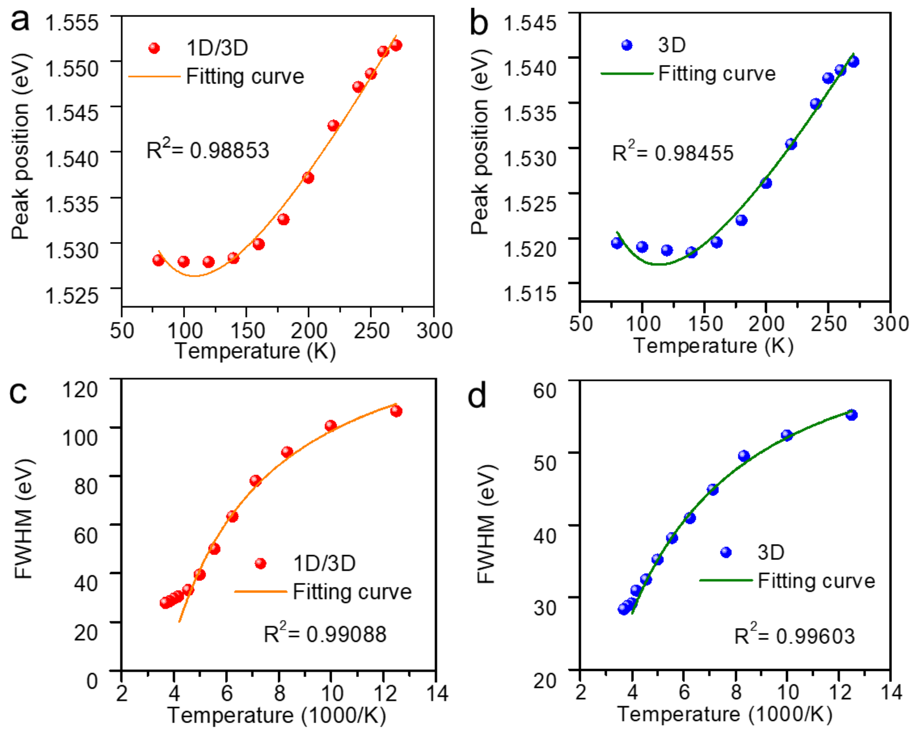

Figure S26. (a, b) peak position (bandgap) and (c, d) FWHM drift fitting curves of the temperature-dependent PL spectra for 1D/3D and bare 3D perovskite films from 80 to 270 K (Supplementary Fig. 23). The similar E_0 (unrenormalized band gap) values of the 3D and 1D/3D films show that the 1D structure could not change the band gap of 3D structure. The 1D/3D structure has smaller full width at half maximum (FWHM) values width than these of 3D film, which indicated the lower coupling coefficient of exciton-phonon in the 1D/3D films.

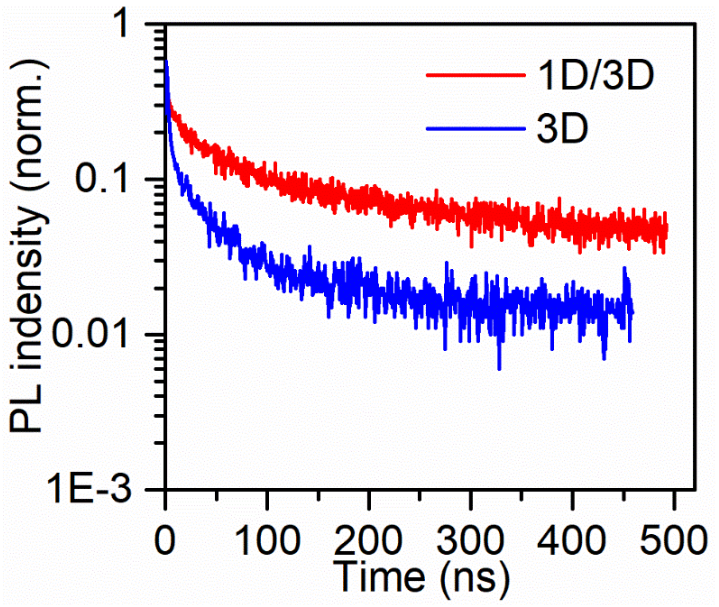

Figure S27. TRPL spectra of perovskite films based on 3D and 1D/3D films.

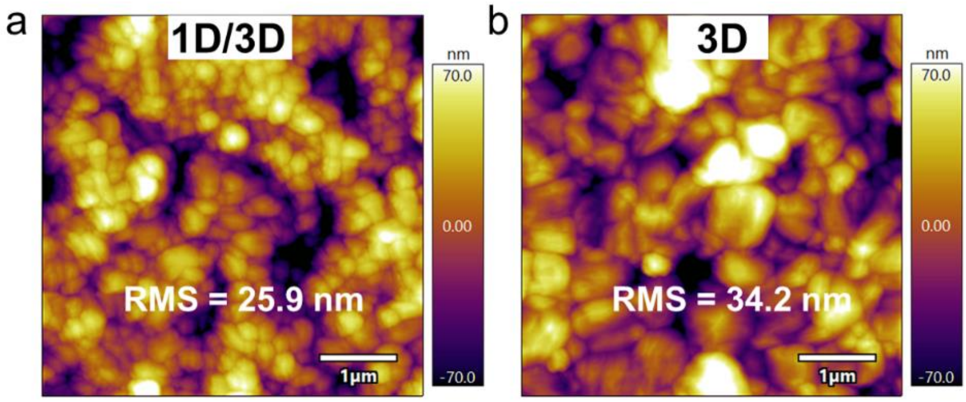

Figure S28. Topography images of (a) 1D/3D film compared with (b) 3D film. RMS represents root mean square roughness.

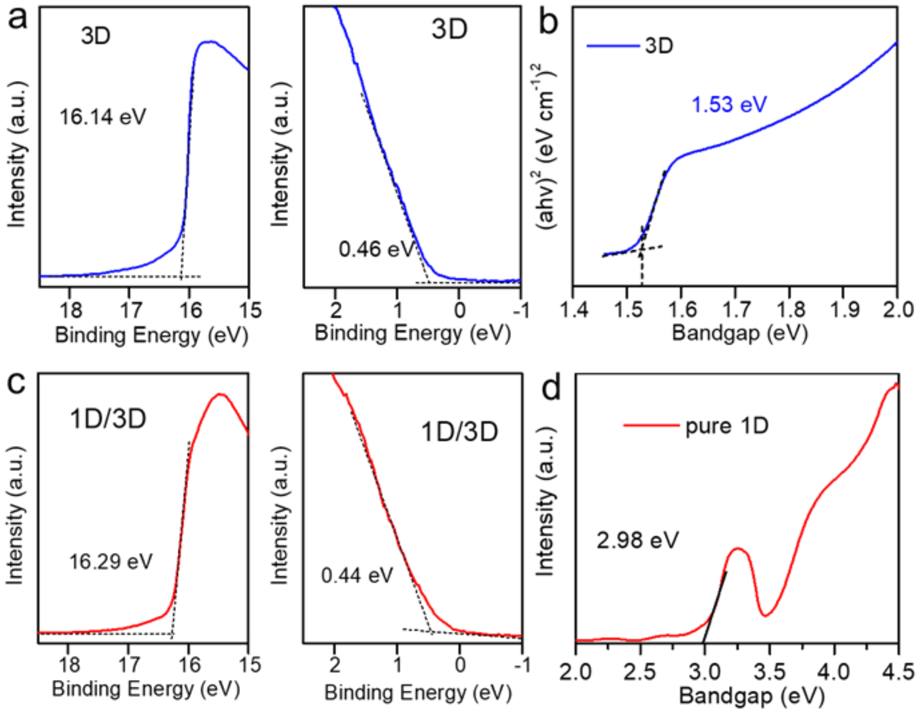

Figure S29. (a, c) Secondary electron cut-off and valence band regions of UPS spectra of the 3D and 1D/3D films. (b, d) Optical bandgap estimation by the Tauc plots for the 3D and 1D films.

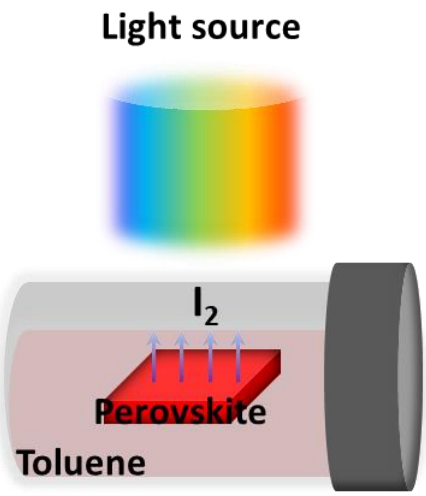

Figure S30. Schematic illustration of the iodine-loss experimental setup.

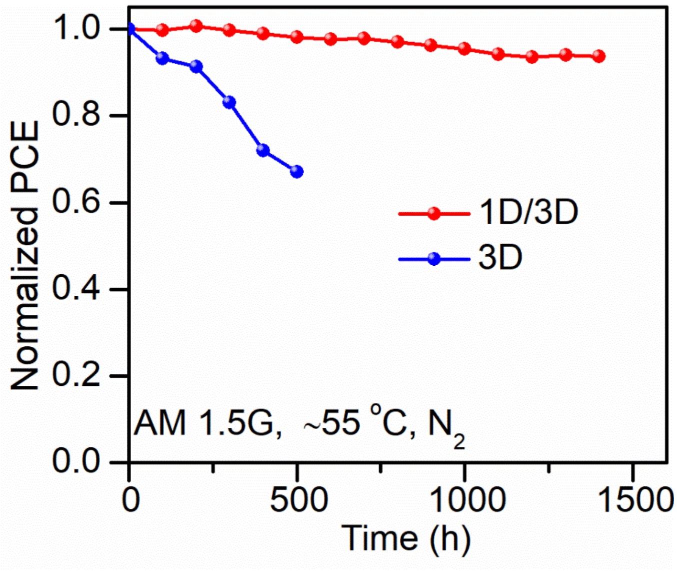

Figure S31. Open-circuit light stability of the 1D/3D and 3D PSCs (0.12 cm^2 of active area) under AM 1.5G illumination (~55 °C, N_2).

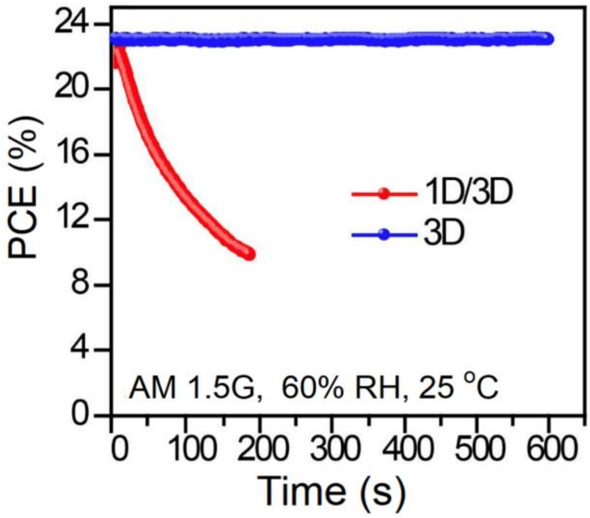

Figure S32. Stabilizing PCE output of the 1D/3D and 3D PSCs (0.12 cm^2 of active area) under AM 1.5G illumination (~25 °C, ～60%RH, air) without any encapsulation.

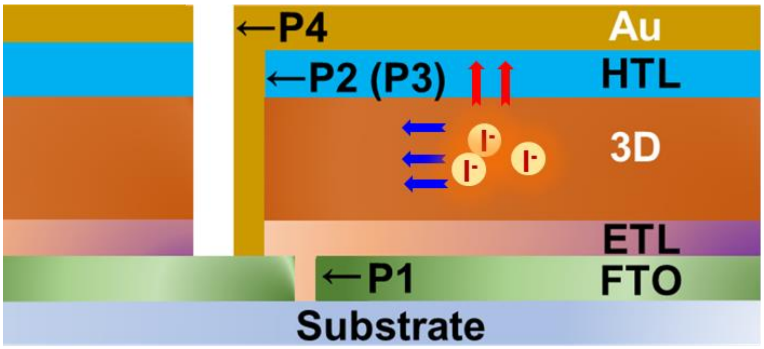

Figure S33. Illustration of the iodide diffusion process (red and blue arrows) in the 3D PSC modules. The iodide could diffuse at the horizontal interconnection and vertical direction.

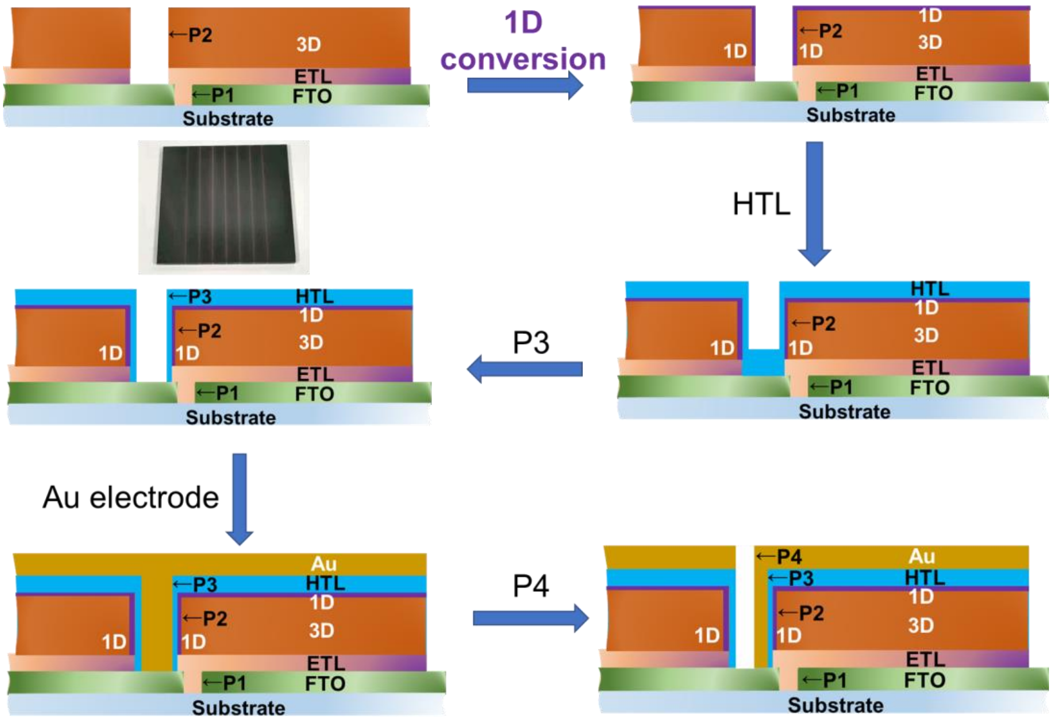

Figure S34. Schematic of the laser-scribing (P1, P2, P3 and P4) process for PSC modules. Inset shows the 36-cm^2-area P2-scribed perovskite film.

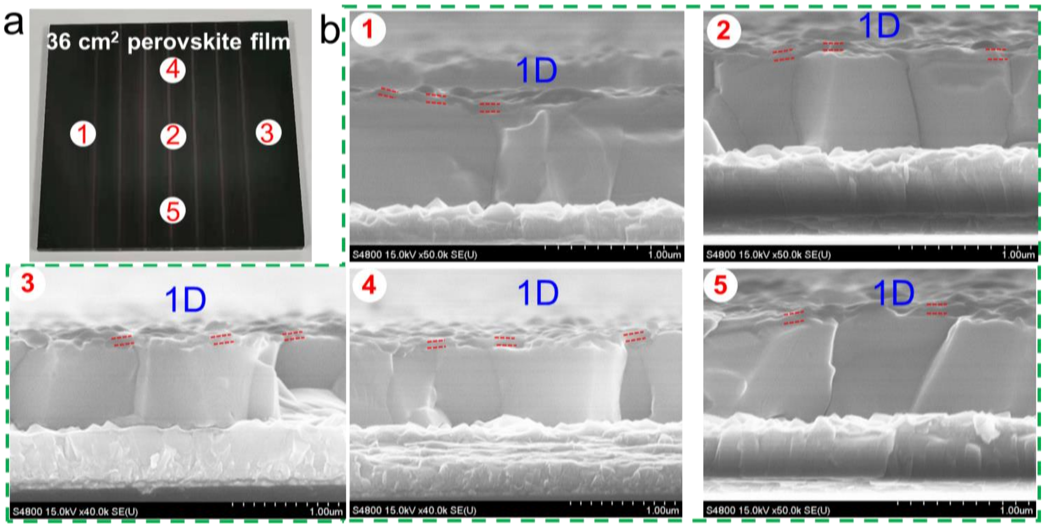

Figure S35. (a) Photo of the 36-cm^2-area perovskite film. (b) The corresponding SEM images of the five regions of 1D/3D perovskite films.

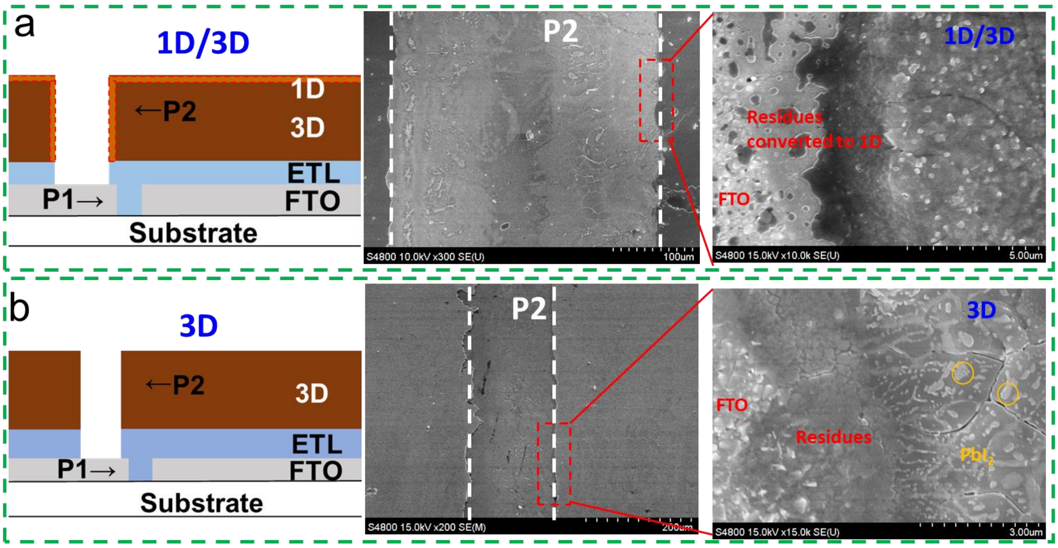

Figure S36. Schematic structure and SEM images of P2 line region on (a) 1D/3D and (b) 3D perovskite films. In the side structure of the P2 line, laser scribing always produced perovskite residues or decomposition products (PbI_2, etc.), and the exposed side perovskite tends to further partially decompose into PbI_2 impurities, while ipr-I post-treatment could enable the forming of 1D structure in the side surface of the perovskite and PbI_2 residue.

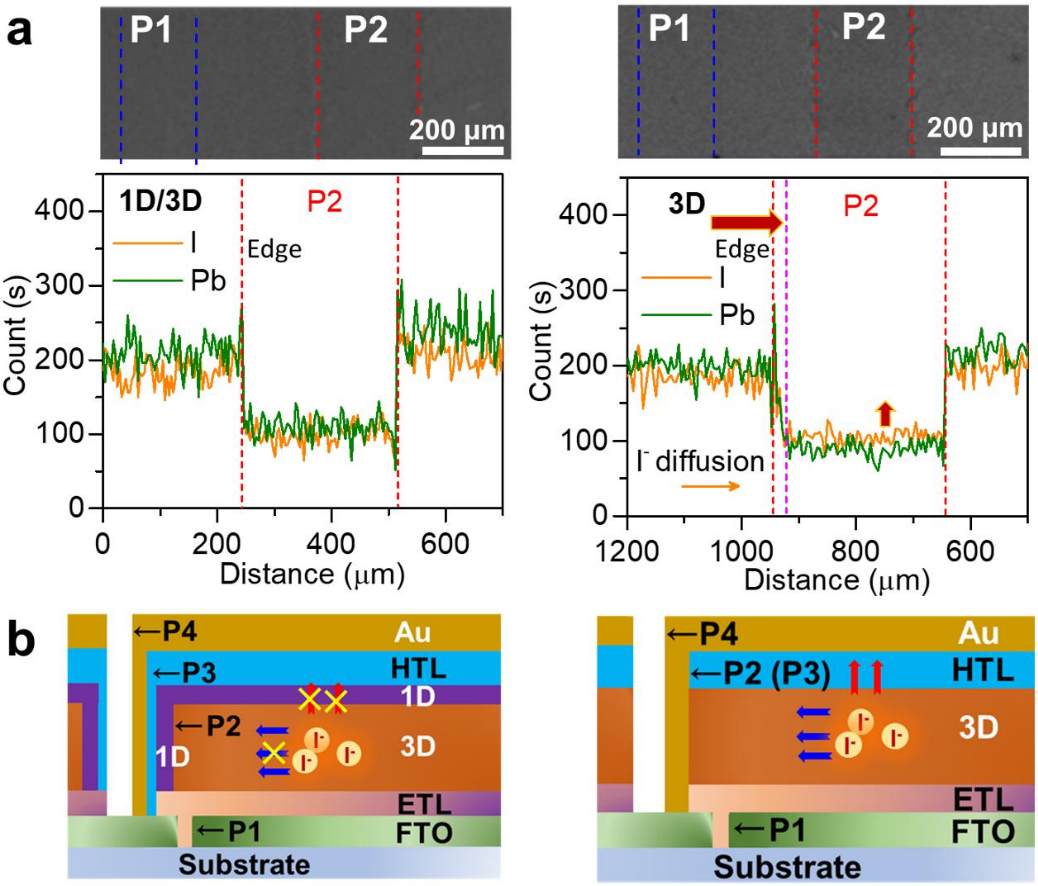

Figure S37. (a) SEM images and the elements distribution for I and Pb measured by EDS line scanning for the lateral structure of 3D and 1D/3D films-based modules. (b) Illustration of the

iodide diffusion process (red and blue arrows) in the 3D and 1D/3D modules. In the fabricated modules, the lateral configuration consists of P1, P2 (P3) and P4 lines, which can interconnect with each subcell. Therefore, the possible iodide diffusion channel should be the P1-P2 interconnection. The SEM-EDS measurements were conducted to probe the iodide distribution in the lateral structure of modules preheated at a temperature of 55 ^{o}C under AM 1.5G illumination and N_2 atmosphere conditions for 100 h. In the P2 line, we can find that the distributed amount of iodide in the 3D module is higher than that in the 1D/3D module, while the lead element has negligibly difference between the two modules. In the P2 line edge, we can see that the iodide and lead elements slightly shifted to P2 line from P1 line. These results indicate that 1D also has effectively suppress the lateral and vertical iodide diffusion within the device while in the series connection.

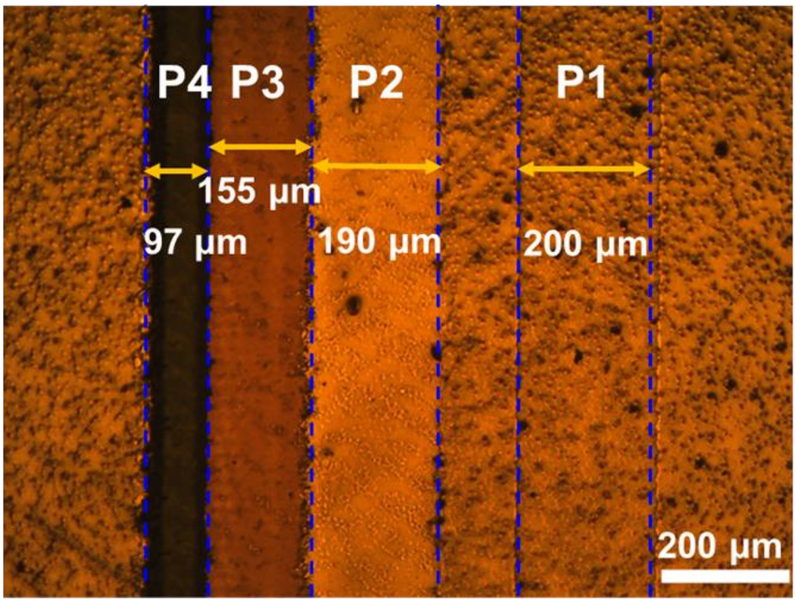

Figure S38. OM image of the 36-cm^2 solar module showing the laser-scribed P1, P2, P3 and P4 lines and related parameters

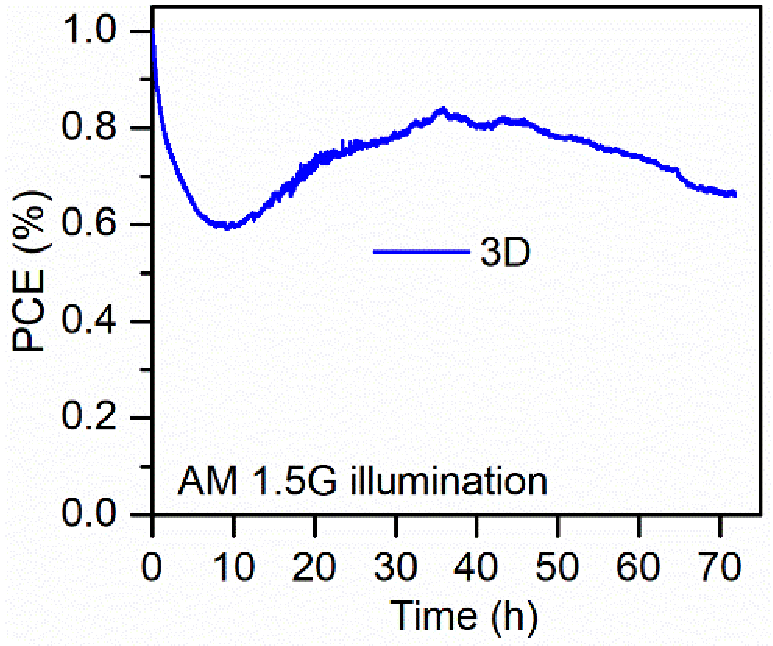

Figure S39. Operation stability of the encapsulated 36-cm^2-area 3D PSC module under maximum power point tracking with AM 1.5G irradiation under 55 ^{o}C and 60% RH.

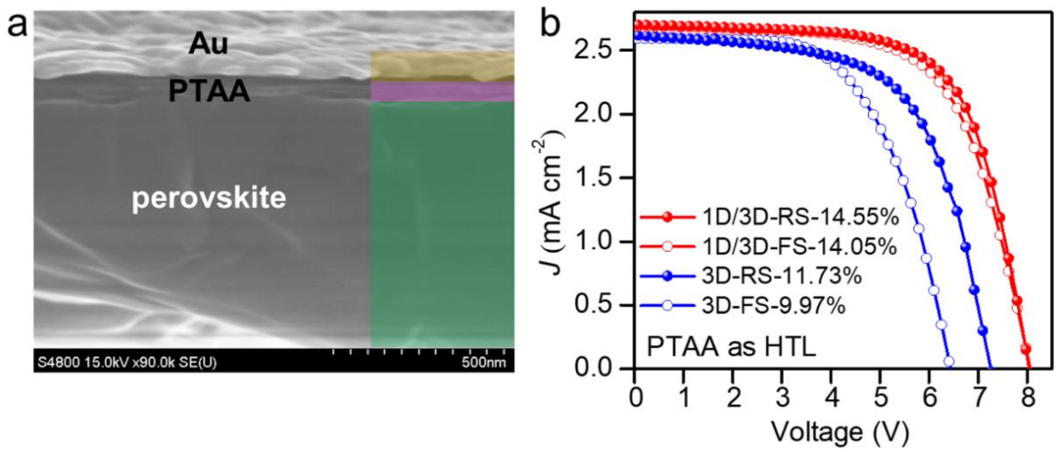

Figure S40. (a) Cross-sectional SEM image of the typical device structure using the PTAA as HTL. (b) J−V curves of the 1D/3D and 3D modules (18 cm^2 of active area) based on PTAA as HTL for "double 85" test.

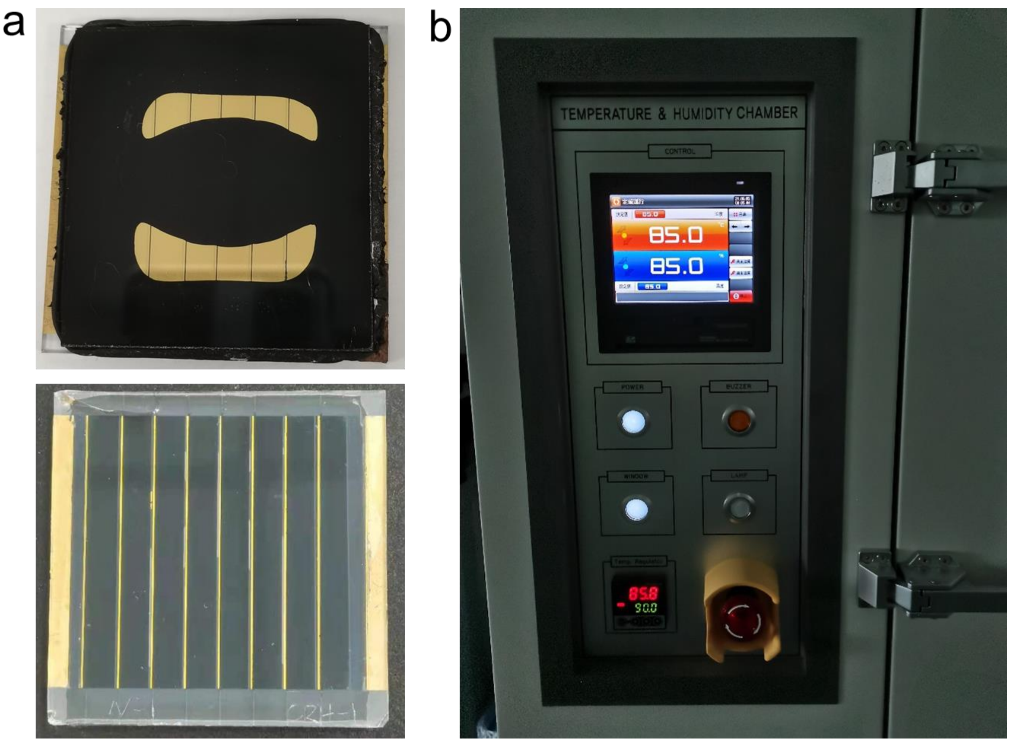

Figure S41. (a) Front and back digital photos of the sealed PSC module (36-cm^2 area) with polyisobutylene (PIB) (HelioSeal PVS101). (b) Set-up and setting parameters of the test chamber.

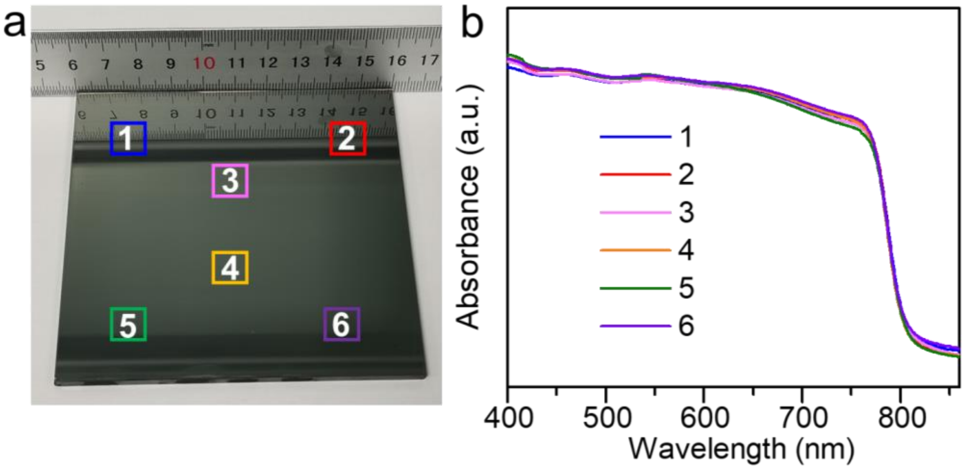

Figure S42. (a) Photo of a large-area (100 cm^2) 1D/3D perovskite film and (b) the corresponding absorption spectra from the six different spots within the film.

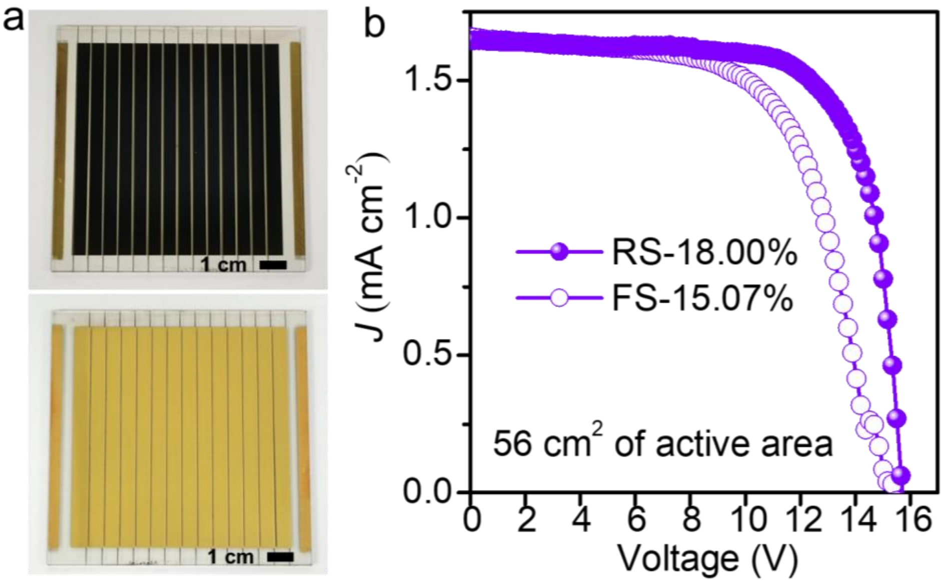

Figure S43. (a) Front and back-view photos of the representative 100 cm^2 area clean-edged PSC modules. (b) J−V characteristics of the 100-cm^2 area PSC module with 1D/3D perovskite films.

Table S1. Crystal data and structure refinement for $mePbI_3$.

CCDC number	2129784
Empirical formula	$C_9H_{11}I_3N_2Pb$
Formula weight	734.05
Temperature/K	100.00(10)
Crystal system	orthorhombic
Space group	*Pbca*
a/Å	16.5187(5)
b/Å	7.8076(2)
c/Å	23.8777(7)
α/°	90
β/°	90
γ/°	90
Volume/Å^3	3079.54(15)
Z	8
ρ_{calc}g/cm^3	3.167
F(000)	2557
Radiation	CuK\a (λ = 1.54184)
2Θ range for data collection/°	4.5560 to 63.5070
Index ranges	$-19 \leq h \leq 12$, $-9 \leq k \leq 8$, $-27 \leq l \leq 26$
Completeness	99.4%
Reflections collected	2503
Independent reflections	2227
Data/restraints/parameters	2503 /72/139
Goodness-of-fit on F^2	1.120
Final R indexes [I>=2σ (I)]	R_1 = 0.0770, wR_2 = 0.1851
Final R indexes [all data]	R_1 = 0.0803, wR_2 = 0.1925
Largest diff. peak/hole / e Å^{-3}	4.00/-1.85

Table S2. Atomic position for $mePbI_3$.

NO.	ELEM.	SYMBOL	X/A	Y/B	Z/C
**1**	Pb	Pb00	0.24821(2)	0.88190(5)	0.56499(2)
**2**	I	I002	0.32222(4)	0.63693(8)	0.65811(3)
**3**	I	I003	0.31271(4)	0.64159(8)	0.46587(3)
**4**	I	I004	0.40588(4)	1.11935(8)	0.57042(3)
**5**	N	N005	0.3668(5)	0.1123(11)	0.3485(4)
**6**	N	N006	0.4443(5)	0.2816(12)	0.3975(3)
**7**	C	C007	0.3841(6)	0.2517(14)	0.3146(4)
**8**	C	C008	0.4032(6)	0.1368(13)	0.3971(5)
**9**	H	H008	0.4002	0.0615	0.4272
**10**	C	C009	0.4954(7)	0.3491(14)	0.4419(4)
**11**	H	H00A	0.4622	0.3839	0.4729
**12**	H	H00B	0.5252	0.446	0.4282
**13**	H	H00C	0.5325	0.2621	0.454
**14**	C	C00A	0.3595(6)	0.2902(15)	0.2595(4)

**15**	H	H00D	0.3267	0.2171	0.2387
**16**	C	C00B	0.4321(6)	0.3619(12)	0.3453(4)
**17**	C	C00C	0.4363(6)	0.5578(15)	0.2704(4)
**18**	H	H00E	0.4526	0.6616	0.255
**19**	C	C00D	0.3878(6)	0.4454(15)	0.2383(4)
**20**	H	H00F	0.3744	0.476	0.2018
**21**	C	C00E	0.3139(7)	-0.0300(16)	0.3334(5)
**22**	H	H00G	0.2599	0.0117	0.3277
**23**	H	H00H	0.3138	-0.1132	0.363
**24**	H	H00I	0.3333	-0.0823	0.2996
**25**	C	C00F	0.4600(5)	0.5168(15)	0.3244(4)
**26**	H	H00J	0.4929	0.589	0.3454

Table S3. Crystal data and structure refinement for $etPbI_3$.

CCDC number	2129781
Empirical formula	$C_{11}H_{15}I_3N_2Pb$
Formula weight	763.14
Temperature/K	100.01(10)
Crystal system	monoclinic
Space group	$P2_1/c$
a/Å	10.7855(2)
b/Å	7.55750(10)
c/Å	21.3050(4)
α/°	90
β/°	93.579(2)
γ/°	90
Volume/Å^3	1733.21(5)
Z	4
ρ_{calc}g/cm^3	2.925
F(000)	1344
Radiation	MoK\a (λ = 0.71073)
2Θ range for data collection/°	3.397 to 27.842
Index ranges	$-14 \leq h \leq 13$, $-9 \leq k \leq 9$, $-24 \leq l \leq 27$
Completeness	99.52%
Reflections collected	3601
Independent reflections	3154
Data/restraints/parameters	3601/0/156
Goodness-of-fit on F^2	1.059
Final R indexes [I>=2σ (I)]	$R_1 = 0.0226$, $wR_2 = 0.0471$
Final R indexes [all data]	$R_1 = 0.0291$, $wR_2 = 0.0498$
Largest diff. peak/hole / e Å^{-3}	0.92/-1.11

Table S4. Atomic position for $etPbI_3$.

NO.	ELEM.	SYMBOL	X/A	Y/B	Z/C
**1**	Pb	Pb01	1.00067(2)	0.44373(2)	0.24832(2)
**2**	I	I002	0.92881(3)	0.69915(3)	0.13212(2)
**3**	I	I003	0.83669(3)	0.68695(3)	0.33420(2)

**4**	I	I004	1.23483(3)	0.69173(3)	0.28466(2)
**5**	N	N005	0.4252(3)	0.7027(4)	0.60861(17)
**6**	N	N006	0.2896(3)	0.8083(4)	0.53680(17)
**7**	C	C007	0.5531(5)	0.7772(6)	0.4330(2)
**8**	H	H007	0.5782	0.7962	0.3926
**9**	C	C008	0.6089(4)	0.6801(5)	0.5398(2)
**10**	H	H008	0.6667	0.637	0.5702
**11**	C	C009	0.4016(4)	0.7849(5)	0.5079(2)
**12**	C	C00A	0.4314(5)	0.8161(5)	0.4457(2)
**13**	H	H00A	0.3738	0.8597	0.4152
**14**	C	C00B	0.4868(4)	0.7179(5)	0.5529(2)
**15**	C	C00C	0.6386(5)	0.7111(6)	0.4783(2)
**16**	H	H00C	0.7187	0.6866	0.4671
**17**	C	C00D	0.3082(4)	0.7569(5)	0.5965(2)
**18**	H	H00D	0.2478	0.7586	0.6258
**19**	C	C00E	0.5561(5)	0.7902(6)	0.7014(2)
**20**	H	H00B	0.6193	0.828	0.6745
**21**	H	H00E	0.5943	0.7467	0.7403
**22**	H	H00F	0.5032	0.8885	0.7098
**23**	C	C00F	0.1031(5)	0.7126(7)	0.4738(3)
**24**	H	H00G	0.1533	0.6638	0.4424
**25**	H	H00H	0.0258	0.7534	0.4542
**26**	H	H00I	0.0873	0.6233	0.5043

Table S5. Crystal data and structure refinement for $iprPbI_3$.

CCDC number	2129783
Empirical formula	$C_{13}H_{19}I_3N_2Pb$
Formula weight	791.19
Temperature/K	100.00(10)
Crystal system	monoclinic
Space group	*C*2/*m*
a/Å	15.4612(4)
b/Å	15.1484(3)
c/Å	8.24829(18)
α/°	90
β/°	99.834(2)
γ/°	90
Volume/Å^3	1903.47(8)
Z	4
ρ_{calc}g/cm^3	2.761
F(000)	1408
Radiation	CuK\a (λ = 1.54184)
2Θ range for data collection/°	4.1170 to 64.9600
Index ranges	$-18 \leq h \leq 18$, $-17 \leq k \leq 16$, $-7 \leq l \leq 9$
Completeness	100.00%
Reflections collected	1660
Independent reflections	1632
Data/restraints/parameters	1660/12/95

Goodness-of-fit on F^2	1.122
Final R indexes [I>=2σ (I)]	R_1 = 0.0453, wR_2 = 0.1268
Final R indexes [all data]	R_1 = 0.0460, wR_2 = 0.1278
Largest diff. peak/hole / e Å^{-3}	2.77/-3.58

Table S6. Atomic position for iprPbI$_3$.

NO.	ELEM.	SYMBOL	X/A	Y/B	Z/C
**1**	Pb	Pb01	1	0.5	0.5
**2**	Pb	Pb02	1	0.5	0
**3**	I	I003	0.83561(4)	0.5	0.69413(9)
**4**	I	I004	0.91556(3)	0.36065(3)	0.22206(6)
**5**	N	N005	0.6357(4)	0.4265(4)	0.2722(9)
**6**	C	C006	0.6528(6)	0.2807(6)	0.1486(11)
**7**	H	H00A	0.5904	0.2763	0.1135
**8**	H	H00B	0.6771	0.2228	0.1709
**9**	H	H00C	0.6787	0.3079	0.0633
**10**	C	C007	0.6718(5)	0.3362(5)	0.3031(10)
**11**	H	H007	0.7357	0.3417	0.3328
**12**	C	C008	0.6849(8)	0.5	0.2903(15)
**13**	H	H008	0.746	0.5	0.3126
**14**	C	C009	0.4703(5)	0.4061(5)	0.2097(11)
**15**	H	H009	0.4701	0.3447	0.2077
**16**	C	C00A	0.5479(5)	0.4540(5)	0.2397(10)
**17**	C	C00B	0.6388(5)	0.2971(6)	0.4481(11)
**18**	H	H00D	0.6572	0.3333	0.5433
**19**	H	H00E	0.6622	0.2387	0.4684
**20**	H	H00F	0.5759	0.2944	0.4254
**21**	C	C00C	0.3940(5)	0.4538(5)	0.1832(11)
**22**	H	H00G	0.3407	0.4239	0.1647

Table S7. Crystal data and structure refinement for hexylPbI$_3$.

CCDC number	2129782
Empirical formula	$C_{19}H_{31}I_3N_2Pb$
Formula weight	875.35
Temperature/K	100.01(10)
Crystal system	hexagonal
Space group	$P6_4$
a/Å	22.1013(5)
b/Å	22.1013(5)
c/Å	9.0557(2)
α/°	90
β/°	90
γ/°	120
Volume/Å^3	3830.79(19
Z	6
ρ_{calc}g/cm^3	2.277
F(000)	2400
Radiation	MoK\a (λ = 0.71073)
2Θ range for data collection/°	0.71073 to 27.948
Index ranges	$-27 \leq h \leq 28$, $-27 \leq k \leq 27$, $-11 \leq l \leq 11$
Completeness	99.53%
Reflections collected	5549

Independent reflections	4299
Data/restraints/parameters	5549/7/228
Goodness-of-fit on F^2	1.092
Final R indexes [I>=2σ (I)]	R_1 = 0.0411, wR_2 = 0.0633
Final R indexes [all data]	R_1 = 0.0753, wR_2 = 0.0745
Largest diff. peak/hole / e Å^{-3}	1.88/-1.51

Table S8. Atomic position for hexylPbI_3.

NO.	ELEM.	SYMBOL	X/A	Y/B	Z/C
**1**	Pb	Pb01	0.28600(3)	0.57722(3)	0.17232(5)
**2**	I	I002	0.20434(5)	0.42247(5)	0.13173(9)
**3**	I	I003	0.40324(5)	0.75541(5)	0.20651(9)
**4**	I	I004	0.16330(5)	0.60005(5)	0.04367(10)
**5**	N	N005	0.1405(6)	0.4522(6)	0.6300(12)
**6**	N	N006	0.0590(6)	0.4298(6)	0.7927(12)
**7**	C	C007	0.0677(8)	0.4368(8)	0.4012(16)
**8**	H	H007	0.1026	0.4472	0.332
**9**	C	C008	-0.0389(8)	0.4083(8)	0.6126(16)
**10**	H	H008	-0.0732	0.3998	0.6822
**11**	C	C009	0.0799(7)	0.4374(7)	0.5482(15)
**12**	C	C00A	0.0275(7)	0.4234(7)	0.6511(14)
**13**	C	C00B	0.0205(8)	0.4082(8)	0.9335(14)
**14**	H	H00A	-0.0095	0.4284	0.9417
**15**	H	H00B	0.0534	0.4256	1.0149
**16**	C	C00D	0.0013(8)	0.4203(8)	0.3586(16)
**17**	H	H00D	-0.0092	0.4181	0.2586
**18**	C	C00E	0.2302(8)	0.2748(8)	0.8389(17)
**19**	H	H00C	0.2724	0.3103	0.8865
**20**	H	H00E	0.191	0.2648	0.9028
**21**	C	C00F	0.2210(8)	0.3028(8)	0.6925(17)
**22**	H	H00F	0.2612	0.3144	0.6306
**23**	H	H00G	0.1801	0.2662	0.6432
**24**	C	C00H	0.2347(12)	0.209(1)	0.821(2)
**25**	H	H00H	0.2715	0.2177	0.7525
**26**	H	H00I	0.1911	0.1721	0.7831

Table S9. Photovoltaic parameters of PSCs based on $FA_{0.9}Cs_{0.1}PbI_3$ films (0.12 cm^2 of active area).

Device		J_{sc}/mA·cm^{-2}	V_{oc}/V	FF/%	η/%
1 mg	Reverse	24.61	1.09	77.21	20.71
	Forward	24.65	1.09	74.36	19.98

3 mg	Reverse	25.11	1.11	77.14	21.50
	Forward	25.15	1.11	76.40	21.32
5 mg	Reverse	24.51	1.12	73.55	20.19
	Forward	24.76	1.12	75.06	20.65
Control	Reverse	24.57	1.06	76.46	19.91
	Forward	24.56	1.06	73.01	19.03

Table S10. Photovoltaic parameters of PSCs based on $FAPbI_3$ films (0.12 cm^2 of active area).

Device		J_{sc}/mA·cm^{-2}	V_{oc}/V	*FF*/%	η/%
1D/3D	Reverse	25.77	1.17	80.70	24.31
	Forward	25.47	1.17	79.14	23.57
3D	Reverse	25.39	1.14	76.75	22.33
	Forward	25.20	1.14	76.03	21.87

Table S11. The performance of PSCs with 1D/3D structure in previous reports and this work.

References	Materials	Configuration	Perovskite preparation	PCE (%) (Reverse scan)
**1**	pentafluorophenylethyl ammonium iodide	1D 3D mixed	Anti-solvent	18.0
**2**	2-(1H-pyrazol-1-yl)pyridine	1D 3D mixed	Anti-solvent	18.1
**3**	thiazole ammonium iodide	1D/3D stacked	Anti-solvent	18.97
**4**	guanidinium bromide	1D 3D Mixed	Anti-solvent	20.29
**5**	bipyridine	1D 3D mixed	Anti-solvent	21.18
**6**	pyrrolidine	1D/3D stacked	Anti-solvent	16.65
**7**	pyrrolidinium hydroiodide	1D/3D stacked	Two-step	19.62
**8**	propargylammonium iodide	1D/3D stacked	Two-step	21.19
**9**	trimethylsulfonium chloride	1D/3D stacked	Anti-solvent	16.67
**10**	hydrazinium bromide	1D 3D Mixed	Anti-solvent	21.2
**11**	Bn^+	1D 3D Mixed	Anti-solvent	21.17
**12**	1, 10-phenanthroline	1D/3D stacked	Two-step	23.3
**This work**	Benzimidazolium derives	1D/3D stacked	Post treatment	**24.3**

Table S12. Photovoltaic parameters of PSCs based on $FA_{0.9}Cs_{0.1}PbI_3$ films (0.12 cm^2 of active area).

Device		J_{sc}/mA·cm^{-2}	V_{oc}/V	*FF*/%	η/%
me-I	Reverse	24.83	1.08	74.38	20.11
	Forward	24.62	1.07	74.62	19.65
et-I	Reverse	25.03	1.07	77.10	20.65
	Forward	24.75	1.07	76.04	20.13
ipr-I	Reverse	25.11	1.11	77.14	21.50
	Forward	25.15	1.11	76.40	21.32
hexyl-I	Reverse	24.57	1.09	75.56	20.23
	Forward	24.30	1.10	73.63	19.68
Control	Reverse	24.57	1.06	76.46	19.91

	Forward	24.56	1.06	73.01	19.03

Table S13. Fitting result based on eq.1 and eq.2.

	Γ_0	σ	Γ_{LO}	$h\omega$	E_0
3D	70.528	0.185	58.34	18 meV	1.458
1D/3D	154.214	0.559	28.42	18 meV	1.467

Table S14. Photovoltaic parameters of solar modules (18 cm^2 of active area).

Device		J_{sc}/mA·cm^{-2}	V_{oc}/V	*FF*/%	η/%
1D/3D	Reverse	3.00	8.68	75.39	19.61
	Forward	3.00	8.54	72.62	18.60
3D	Reverse	2.92	8.38	68.74	16.82
	Forward	2.96	8.21	61.33	14.90

Table S15. Photovoltaic parameters of PSCs based on PTAA HTL (18 cm^2 of active area).

Device		J_{sc}/mA·cm^{-2}	V_{oc}/V	*FF*/%	η/%
1D/3D	Reverse	2.70	8.03	67.11	14.55
	Forward	2.69	8.05	64.89	14.05
3D	Reverse	2.62	7.28	61.50	11.73
	Forward	2.62	6.45	59.00	9.97

Table S16. Photovoltaic parameters of solar modules (56 cm^2 of active area).

Device		J_{sc}/mA·cm^{-2}	V_{oc}/V	*FF*/%	η/%
1D/3D	Reverse	1.59	15.71	72.01	18.00
	Forward	1.60	15.31	61.52	15.07

Notes:

The peak position and half-width data for the temperature-dependent photoluminescence (TDPL) spectra were obtained by Gaussian function fitting. The FWHM broadening characteristics can be used to analyze the exciton-phonon coupling mechanism and defect density through the Toyozawa equation (eq.1)

$$\Gamma(T) = \Gamma_0 - \sigma T - \frac{\Gamma_{LO}}{e^{\frac{\hbar\omega_{LO}}{k_B T}} - 1} \quad \text{(eq.1)}$$

Γ_0 represents the contribution of crystal defects and disorder to the scattering of phonons, that is, the contribution of inhomogeneous broadening; $\sigma$is the expansion coefficient of acoustic phonons with temperature; $\Gamma_{LO}$is the exciton-optical phonon coupling coefficient, which represents the uniform broadening term; $\hbar\omega_{LO}$ is the LO phonon energy. The relationship between peak position and temperature can be estimated using a simplified Bose-Einstein band gap model (eq.2):

$$E(T) = E_0 - A\left(\frac{1}{e^{\hbar\omega/k_B T} - 1} - \frac{1}{2}\right) \quad \text{(eq.2)}$$

E_0 represents the band gap value under 0 K conditions, A represents a constant, $h\omega$ is the effective phonon energy.

References

1. Bi DQ*, et al.* High-Performance Perovskite Solar Cells with Enhanced Environmental Stability Based on Amphiphile-Modified CH3NH3PbI3. *Adv. Mater.* **28**, 2910-2915 (2016).
2. Fan JD*, et al.* Thermodynamically Self-Healing 1D-3D Hybrid Perovskite Solar Cells. *Adv. Energy Mater.* **8**, (2018).
3. Gao L*, et al.* Improved Environmental Stability and Solar Cell Efficiency of (MA,FA)PbI_3 Perovskite Using a Wide-Band-Gap 1D Thiazolium Lead Iodide Capping Layer Strategy. *ACS Energy Lett.* **4**, 1763-1769 (2019).
4. Zhang W, Xiong J, Li J, Daoud WA. Guanidinium induced phase separated perovskite layer for efficient and highly stable solar cells. *J. Mater. Chem. A* **7**, 9486-9496 (2019).
5. Ma C*, et al.* High performance low-dimensional perovskite solar cells based on a one dimensional lead iodide perovskite. *J. Mater. Chem. A* **7**, 8811-8817 (2019).
6. Liu P*, et al.* Lattice-Matching Structurally-Stable 1D@3D Perovskites toward Highly Efficient and Stable Solar Cells. *Adv. Energy Mater.* **10**, (2020).
7. Pham ND*, et al.* 1D Pyrrolidinium Lead Iodide for Efficient and Stable Perovskite Solar Cells. *ENERGY TECHNOLOGY* **8**, (2020).
8. Xu AF*, et al.* Promoting Thermodynamic and Kinetic Stabilities of FA-based Perovskite by an in Situ Bilayer Structure. *Nano Lett.* **20**, 3864-3871 (2020).
9. Yang N*, et al.* An in situ cross-linked 1D/3D perovskite heterostructure improves the stability of hybrid perovskite solar cells for over 3000 h operation. *Energy Environ. Sci.* **13**, 4344-4352 (2020).
10. Elsenety MM*, et al.* Stability Improvement and Performance Reproducibility Enhancement of Perovskite Solar Cells Following (FA/MA/Cs)$PbI_{3-x}Br_x$/$(CH_3)_3SPbI_3$

Dimensionality Engineering. *ACS Applied Energy Materials* **3**, 2465-2477 (2020).

11. Yu S, Liu H, Wang S, Zhu H, Dong X, Li X. Hydrazinium cation mixed $FAPbI_3$-based perovskite with 1D/3D hybrid dimension structure for efficient and stable solar cells. *Chem. Eng. J.* **403**, 125724 (2021).
12. Zhan Y*, et al.* Elastic Lattice and Excess Charge Carrier Manipulation in 1D-3D Perovskite Solar Cells for Exceptionally Long-Term Operational Stability. *Adv. Mater.* **33**, (2021).
13. Chen QH, Deng KM, Shen Y, Li L. Stable one dimensional (1D)/three dimensional (3D) perovskite solar cell with an efficiency exceeding 23%. InfoMat **4**, (2022).
